# A Genetic Map for the Only Self-Fertilizing Vertebrate

**DOI:** 10.1534/g3.115.022699

**Published:** 2016-02-09

**Authors:** Akira Kanamori, Yosuke Sugita, Yasufumi Yuasa, Takamasa Suzuki, Kouichi Kawamura, Yoshinobu Uno, Katsuyasu Kamimura, Yoichi Matsuda, Catherine A. Wilson, Angel Amores, John H. Postlethwait, Koushirou Suga, Yoshitaka Sakakura

**Affiliations:** * Division of Biological Science, Graduate School of Science, Nagoya University, Aichi 464-8602, Japan; † Japan Science and Technology Agency (JST) Exploratory Research for Advanced Technology (ERATO) Higashiyama Live-Holonics Project, Nagoya University, Aichi 464-8602, Japan; ** Department of Applied Molecular Biosciences, Graduate School of Bioagricultural Sciences, Nagoya University, Aichi 464-8602, Japan; ‡ Department of Biological Chemistry, College of Bioscience and Biotechnology, Chubu University, Kasugai, Aichi 487-8501, Japan; § Graduate School of Bioresources, Mie University, Tsu, Mie 514-8507, Japan; †† Institute of Neuroscience, University of Oregon, Eugene, Oregon 97403-1254; ‡‡ Graduate School of Fisheries Science and Environmental Studies, Nagasaki University, 852-8521, Japan

**Keywords:** phylogeny by RAD-seq, centromeres and recombination, conserved chromosomes, hermaphrodite, teleost, genetics of sex

## Abstract

The mangrove killifish *Kryptolebias marmoratus*, and its close relative *Kryptolebias hermaphroditus*, are the only vertebrate species known to reproduce by self-fertilization due to functional ovotestis development. To improve our understanding of their genomes, we constructed a genetic map. First, a single F_1_ fish was made by artificial fertilization between *K. marmoratus* and *K. hermaphroditus* strains. F_2_ progeny were then obtained by self-fertilization of the F_1_ fish. We used RAD-seq to query genomic DNAs from the two parental strains, the F_1_ individual and 49 F_2_ progeny. Results identified 9904 polymorphic RAD-tags (DNA markers) that mapped to 24 linkage groups, corresponding to the haploid chromosome number of these species. The total length of the map was 1248 cM, indicating that about one recombination occurred for each of the 24 homologous chromosome pairs in each meiosis. Markers were not evenly distributed along the chromosomes: in all chromosomes, many markers (> 8% of the total markers for each chromosome) mapped to chromosome tips. Centromeres suppress recombination, and this uneven distribution is probably due to the species’ acrocentric chromosomes. Mapped marker sequences were compared to genomic sequences of medaka and platyfish, the next most closely related species with sequenced genomes that are anchored to genetic maps. Results showed that each mangrove killifish chromosome corresponds to a single chromosome of both platyfish and medaka, suggesting strong conservation of chromosomes over 100 million years of evolution. Our genetic map provides a framework for the *K. marmoratus*/*K. hermaphroditus* genome sequence and an important resource for understanding the biology of hermaphroditism.

The mangrove killifish *Kryptolebias marmoratus* (formerly *Rivulus marmoratus*), and its close relative *Kryptolebias hermaphroditus* (formerly *Kryptolebias ocellatus*) (Costa 2011), are the only vertebrate species known to reproduce by self-fertilization (Harrington 1961). A pair of gonads, suspended by a thin mesogonium (gonadal mesentery), consists predominantly of ovarian tissue with a small amount of testicular tissue at the base of the mesogonium (Harrington 1967, 1975; Sakakura *et al.* 2006; Kanamori *et al.* 2006). Ovulated eggs are fertilized in the ovarian cavity by sperm from the same gonad (Harrington 1967; Sakakura *et al.* 2006) and developing embryos are laid mostly within 3 d after fertilization (Harrington 1963). Due to self-fertilization, most loci are homozygous in laboratory strains and even in wild fish (Turner *et al.* 1992; Laughlin *et al.* 1995). Genetic studies of wild fish, however, suggested that rare males outcross with hermaphrodites (Lubinski *et al.* 1995; Mackiewicz *et al.* 2006), thus retaining a low level of genetic diversity over generations. *K. marmoratus*/*K. hermaphroditus* have several remarkable characteristics that provide an opportunity to inform general problems, including phenotypic diversity with clonal genomes, development of ovotestis, cutaneous breathing, functional spermatogenesis and oogenesis within the same gonad, and exceptionally aggressive behaviors against other *K. marmoratus*/*K. hermaphroditus* individuals (see Orlando 2012).

The *K. marmoratus*/*K. hermaphroditus* genome, however, has not been described fully because relatively short contigs obtained from next-generation sequencing are challenging to assemble without a genetic map (Kelley *et al.* 2012; Rhee and Lee 2014). Recently, restriction site associated DNA sequencing (RAD-seq) has been used to make genetic maps with large numbers of DNA markers (Baird *et al.* 2008). This method involves next-generation sequencing of short DNA stretches adjacent to restriction sites (RAD-tags), and is relatively simple and applicable to genetically uncharacterized organisms, including many non-model organisms such as various fish species (Amores *et al.* 2011, 2014; Palaiokostas *et al.* 2013a,b; Recknagel *et al.* 2013; Brieuc *et al.* 2014; Gonen *et al.* 2014). To make a genetic cross from generally self-fertilizing species, we used artificial fertilization between *K. marmoratus* strain DAN as a paternal parent and *K. hermaphroditus* strain PAN-RS as a maternal parent (Nakamura *et al.* 2008). We generated RAD-tags from both parental strains, an F_1_ fish, and 49 F_2_ individuals produced by self-fertilization of the F_1_ fish. From 9904 RAD-tags with single nucleotide polymorphisms (SNPs) between parental strains, we obtained a genetic map with 24 linkage groups, which corresponds to the known number of haploid chromosomes in both *K. marmoratus* and *K. hermaphroditus* (Scheel 1972; Sola *et al.* 1997). This genetic map provides a genomic tool for: 1) assembly of genome sequence scaffolds obtained from next-generation sequencing (Sucar *et al*. 2016); 2) positional cloning of candidate genes from mutagenesis screening; and 3) mapping of QTL from wild fish or recombinant inbred lines, which can be obtained easily due to self-fertilization (Nakamura *et al.* 2008), and therefore provides an important resource for understanding the biology of hermaphroditism.

## Materials and Methods

### Mapping cross

Artificial fertilization of sperm from a single DAN (*K. marmoratus*) fish and eggs from a single PAN-RS (*K. hermaphroditus*) individual produced a single F_1_ fish, and self-fertilization of the F_1_ individual produced the F_2_ progeny of the mapping cross (Nakamura *et al.* 2008). The clonal strains, PAN-RS and DAN, were originally collected near Bocas del Tora, Republic of Panama, and Dangriga, Belize, respectively, and have been kept at the Aquaculture Biology Laboratory in Nagasaki University. PAN-RS and DAN were compared to three strains: a *K. marmoratus* strain VOL (Volusia County, Florida), a *K. hermaphroditus* strain HY (a gift from Higashiyama Zoo, Nagoya, Japan), and a sister species *Kryptolebias caudomarginatus*, which is gonochoristic (having separate male and female individuals) and were obtained from Higashiyama Zoo, Nagoya, Japan. These strains have been maintained at Nagoya University since 2004. The Animal Care and Use Committee of Nagoya University approved all husbandry and experimental procedures in the present study.

### RAD sequencing

Genomic DNA was extracted from frozen or fresh tissue with the DNeasy Blood and Tissue DNA kit (QIAGEN), digested with high-fidelity *Sbf*I (New England Biolabs), ligated with 5-nucleotide bar-coded adaptors, multiplexed, sonicated, blunted, ligated with another Y-shaped adaptor, and PCR amplified (for details, see Amores *et al.* 2011). To remove short DNA fragments or nucleotides, magnetic beads (Agencourt AMPure XP) were used instead of agarose gel purification as originally described. To generate RAD-tags, approximately 40 samples from equal starting amounts of genomic DNA were mixed at 65 ng/20 μl (10 nM) and sequenced on an Illumina HiSequation 2500 with 101 bp single-end reads (75 samples in two lanes).

### Genotyping by STACKS

RAD-tag sequences were quality-filtered with the process_radtags module of STACKS software (http://creskolab.uoregon.edu/stacks/, Catchen *et al.* 2011). Low quality sequences (average phred score less than 10 over a 15-nucleotide sliding window) and reads with uncalled bases were discarded, as were sequences missing barcodes or *Sbf*I restriction sites. A total of 75 samples yielded, on average, 63,000 tags per sample and 61 retained reads per tag. We used STACKS to assemble retained sequences into tags and to call genotypes for each tag. For phylogenetic tree construction, sequences from *K. marmoratus* (DAN and VOL), *K. hermaphroditus* (PAN-RS and HY), and *K. caudomarginatus* (Kc) were analyzed as populations with the following parameters: −M 4 −n 4 –m 10 (Catchen *et al.* 2013). Because genomic DNAs from the parents were unavailable, we reconstructed parental genotypes from five DAN (*K. marmoratus*) and six PAN-RS (*K. hermaphroditus*) individuals. Because both DAN and PAN-RS are isogenic by many rounds of self-fertilization, we assumed that any individuals of DAN or PAN-RS have genotypes identical to the parental individuals. For F_2_ mapping, we created a STACKS catalog from these parental strains, which we used to call genotypes in the F_1_ and F_2_ progeny with the following parameters: −M 4 −n 4 –m 10 −P 3, and default genotyping parameters; five reads were required to call a homozygous genotype, a minimum minor allele frequency of 0.1 was required to call a heterozygote, and a maximum minor allele frequency of 0.05 was required to call a homozygote. Sequences were deposited in the SRA (Sequence Read Archive, http://www.ncbi.nlm.nih.gov/sra) under the accession number SRP060021.

### Phylogenetic analysis

We RAD-sequenced genomic DNA from six individuals of PAN-RS, five of DAN, and two each of HY, VOL and *K. caudomarginatus* (Kc). Approximately 58,000 tags were recovered from each individual. Tags were discarded if they met the following criteria: 1) tags with genotypes missing in ≥ 3 individuals for PAN-RS, ≥ 2 for DAN, or ≥ 1 for HY, VOL, and Kc were discarded (1–3%); 2) polymorphic tags within a strain were discarded (0.5–1%) because they likely represented repetitive sequences; 3) polymorphic tags within a single individual were discarded (0.5–1%) because they likely represented repetitive sequences. About 57,000 homozygous tags per strain were retained for analysis because they likely represented unique sequences in each genome (Supplemental Material, Table S1). For mitochondrial sequences, a part of the 12S rRNA-tRNA-16S rRNA (2.1 kb) was PCR amplified with a primer pair described by Hrbek and Larson (1999) (L1090 and H3058) and cycle-sequenced with the same primers by BigDye terminator ver3.1 (Life Technologies) and an ABI 3100 sequencer (Applied Biosystems). Parts of the 12S (706 bases) and 16S rRNAs (725 bases) were combined for alignment. GenBank accession numbers are as follows: KP998185-89 for 16S DAN, HY, Kc, PAN-RS, and VOL; KP998190-94 for 12S DAN, HY, Kc, PAN-RS, and VOL. Phylogenetic trees were constructed with MEGA 6 (Tamura *et al.* 2013) with 10,000 bootstrap replicates.

### Linkage mapping

We recovered 60,662 and 62,141 tags for DAN and PAN-RS, respectively, and 56,397 of them were shared between DAN and PAN-RS. 579 tags were heterozygous within either DAN or PAN-RS and were removed because they likely represented repetitive elements. Finally, 45,499 tags were not polymorphic between DAN and PAN-RS, leaving 10,319 tags polymorphic between DAN and PAN-RS (hereafter called markers). Heterozygosity of these markers was about 97% for the F_1_ and 50% for all F_2_ individuals, except one F_2_ individual with about 11%. This F_2_ individual was most likely derived from a sampling error of a later generation than F_2_ and removed from further analysis. An additional seven F_2_ progeny were omitted because they were missing genotypes for more than 800 markers, leaving a total of 49 F_2_. Next, markers showing heavy segregation distortion (*P* < 0.0001, 12 markers) and markers missing genotypes from more than seven F_2_ individuals (370 markers) were excluded because these markers could not be mapped with confidence. The final F_2_ genotyped mapping panel consisted of 49 F_2_ with 9937 markers (map49F2_42.txt in File S3). The panel was processed by genetic mapping software written in PHP (linkage.class.php in File S3). This simple script classifies cosegregating markers into bins (map locations of markers having identical genotype patterns in the panel), calculates recombination rates among all bins, and links bins using the nearest neighboring method judged by recombination rates. Further details of the program are summarized as MappingOutline.pdf in File S3. We disregarded missing genotypes (0–7 for 49 F_2_) and grouped markers by identical patterns of remaining markers. If a marker could belong to 2 (116 markers) or 3 bins (3 markers), it was arbitrarily assigned to one of the bins. Our mapping algorithm was compared to those of two established mapping programs. First, the F_2_ genotyped panel (map49F2_42.txt) was reanalyzed using JoinMap 4.1 (Van Ooijen 2011) for linkage analysis using the “Independence LOD” parameter under the “Population Grouping” tab with a minimum LOD value of 12. After the initial grouping to individual LGs, marker ordering was performed using the Maximum Likelihood algorithm with default parameters. Second, the final genotyped panel (File S1) was reanalyzed with AntMap, which is optimized for large numbers of bins by the “Ant Colony Optimization” method (http://lbm.ab.a.u-tokyo.ac.jp/~iwata/antmap/, Iwata and Ninomiya 2006).

### Sequence comparisons

Mapped marker sequences (total 9904, File S2) were compared to the genomic sequences of medaka (ftp://ftp.ensembl.org/pub/release-76/fasta/oryzias_latipes/dna/Oryzias_latipes.MEDAKA1.dna.chromosome.$G.fa.gz) and platyfish (http://genome.uoregon.edu/xma/v1.0/xma_washu_4.4.2-jhp_1.0.fa.gz) by blastn with default parameters: gap opening penalty = 5, gap extension penalty = 2, nucleic match = 1, nucleic mismatch = −3, expectation value = 10.0, word size = 11 (Altschul *et al.* 1990).

### Cell culture and chromosome preparation

Adult HY (*K. hermaphroditus*) were anesthetized by MS-222 (Sigma), and fins were dissected and minced for primary culture of fibroblasts as described in Uno *et al.* (2013). Cells were cultured at 26° in a humidified atmosphere of 5% CO_2_ in air. After treatment with colcemid (80 ng/ml) for 90 min, the primary cultured fibroblast cells were harvested, and chromosome preparations were made using an air-drying method following a standard protocol.

### Data availability

RAD-tag sequences used in the present study were deposited in the SRA (Sequence Read Archive, http://www.ncbi.nlm.nih.gov/sra) under the accession number SRP060021. The genetic map shown in Figure 2 was constructed from the final F2 genotyped panel consisted of 49 F2 with 9937 markers (map49F2_42.txt in the supplementary File S3) by mapping software written in PHP (scripts and instructions can be found in File S3). The final map data and marker sequences are provided as File S1 and File S2, respectively.

## Results and Discussion

### Phylogenetic relationships among Kryptolebias strains

**Figure 1:**
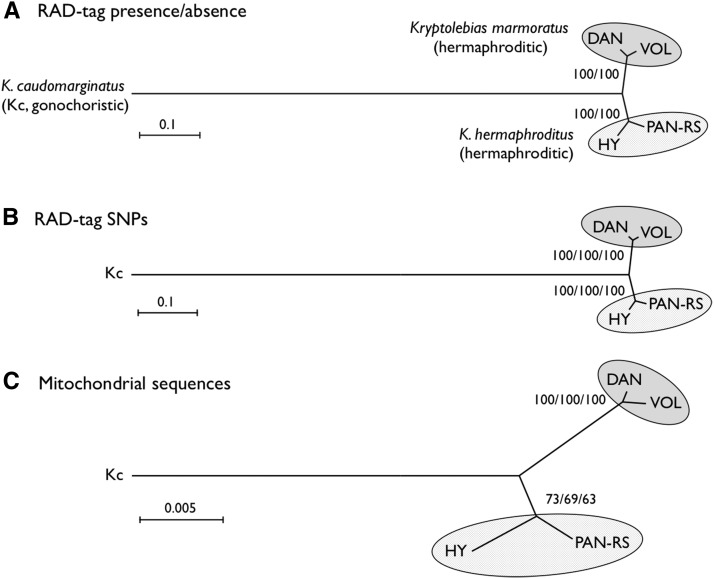
Phylogenetic relationships of two hermaphroditic mangrove killifish species, *Kryptolebias marmoratus* (strains DAN and VOL) and *K. hermaphroditus* (strains PAN-RS and HY), and a sister gonochoristic species, *K. caudomarginatus* (Kc). Trees were constructed by neighbor-joining (NJ) based on the presence or absence of RAD-tags (A), concatenated SNPs (53,544 polymorphic, informative bases) identified from RAD-tags (B), and from mitochondrial DNA sequences (a part of 12S and 16S rRNA combined, total 1431 bases) (C), respectively. The tree topology did not change when analyzed with maximum likelihood (ML) or maximum parsimony (MP) methods (data not shown). Numbers at the nodes represent percentage recovery of those nodes per 10,000 bootstrap replicates (NJ/MP for A and NJ/ML/MP for B and C). Scale bars indicate genetic distance.

We used strains DAN (*K. marmoratus*) and PAN-RS (*K. hermaphroditus*) as parents for our F_2_ mapping panel. Analysis of 32 microsatellite loci has shown that these two strains are distantly related among hermaphroditic mangrove killifish (Tatarenkov *et al.* 2010). To determine whether this conclusion holds for our strains and is robust with our much larger dataset, we tested several strains by RAD-seq analyses. An advantage of RAD-seq is the rapid identification of a large number of SNPs that can be utilized to construct phylogenetic trees (Cui *et al.* 2013; McCluskey and Postlethwait 2015). About 57,000 tags, which probably represent unique sequences in each genome, were obtained from each of the various *K. marmoratus*/*K. hermaphroditus* strains and their gonochoristic sister species, *K. caudomarginatus* (Table S1). More than half of all tags (35,415) were shared by all taxa. The remaining tags were missing from one or more samples, presumably due to strain-specific differences in restriction enzyme recognition sites. To construct phylogenetic trees based on the presence or absence of tags by neighbor-joining (NJ) method, we used the number of absent tags as genetic distance; for the maximum parsimony (MP) method, we used the presence of tags as the derived state. The maximum likelihood (ML) method was not applied because nucleotide sequence status could not be inferred in the absence of tags. The tree topology obtained using NJ (Figure 1A) was identical to that from MP (not shown), demonstrating with high bootstrap values that the *K. marmoratus* strains DAN and VOL group as sisters and the *K. hermaphroditus* strains PAN-RS and HY group as sisters, with *K. caudomarginatus* falling as a distant outgroup. After making a tree using tag presence/absence as characters, we used SNPs in RAD-tags as characters. Among 35,415 tags shared by all five samples, 26,175 contained SNPs that were polymorphic in one or more samples (Table S1). The total SNP count was 53,544 bases because some tags had more than one SNP. We concatenated SNPs and constructed phylogenetic trees by NJ, ML, and MP methods. NJ and ML methods used Kimura-2-Parameter distance. Results showed that the topology of the NJ tree (Figure 1B) was identical to those of the ML and MP trees (not shown), with *K. marmoratus* DAN and VOL as sisters, and *K. hermaphroditus* PAN-RS and HY falling as sisters and with high bootstrap support. Tree topology from the two methods (tag presence/absence or concatenated SNPs) was identical and similar to the tree obtained from conventional mitochondrial sequences analyzed by NJ, ML, and MP methods (Figure 1C shows the NJ tree). These identical topologies and 100% bootstrap values strongly demonstrate the utility of RAD-seq for phylogenetic analyses among related species, as already suggested (Rubin *et al.* 2012; Cariou *et al.* 2013; Cui *et al.* 2013; McCluskey and Postlethwait 2015). A portion of mitochondrial 12S rRNA sequences obtained in this study (316 bases in total) were aligned to published sequences (Hrbek and Larson 1999; Murphy *et al.* 1999; Lee *et al.* 2001; Vermeulen and Hrbek 2005) to make a phylogenetic tree, including sequence data from an outgroup, *Kryptolebias brasiliensis* (Vermeulen and Hrbek 2005) (Figure S1). The resulting tree confirmed the sister relationship of the hermaphroditic species *K. marmoratus*/*K. hermaphroditus* and the gonochoristic species *K. caudomarginatus* (Murphy *et al.* 1999; Vermeulen and Hrbek 2005), as well as the presence of two hermaphroditic species: *K. marmoratus* containing DAN and VOL and *K. hermaphroditus* containing PAN-RS and HY.

### Genetic linkage map

**Figure 2:**
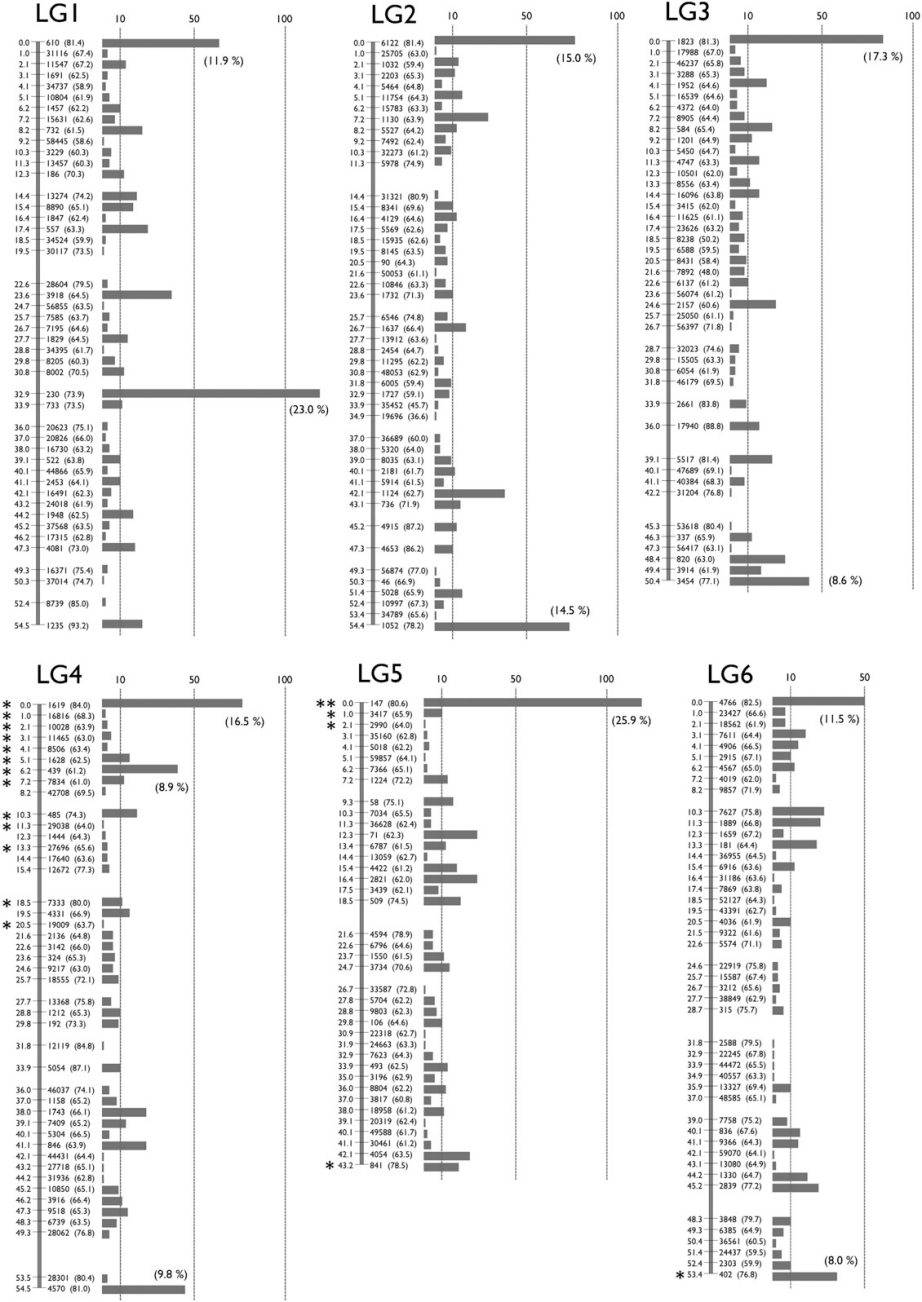

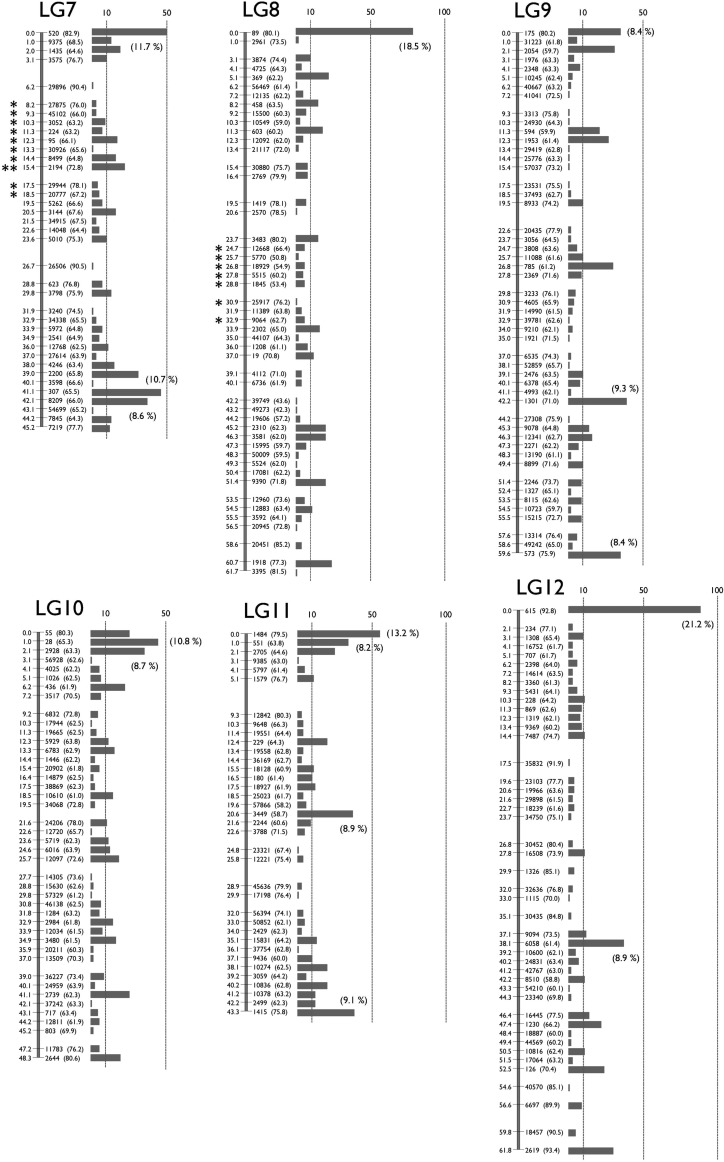

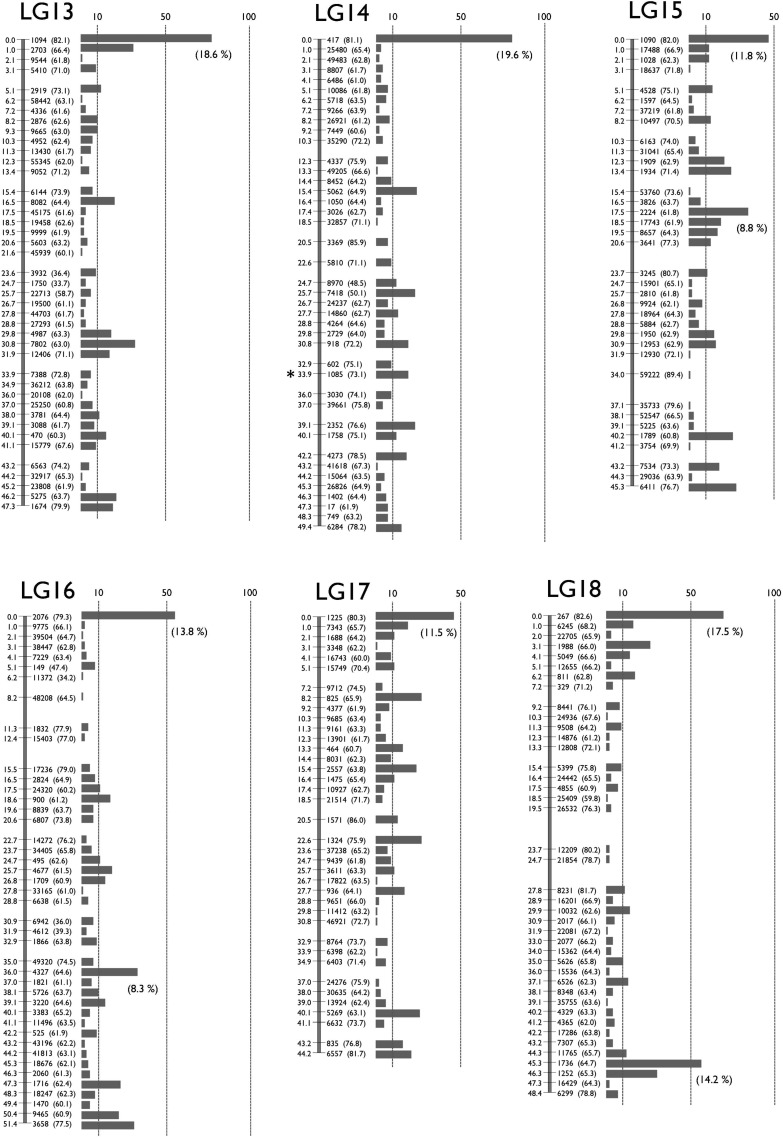

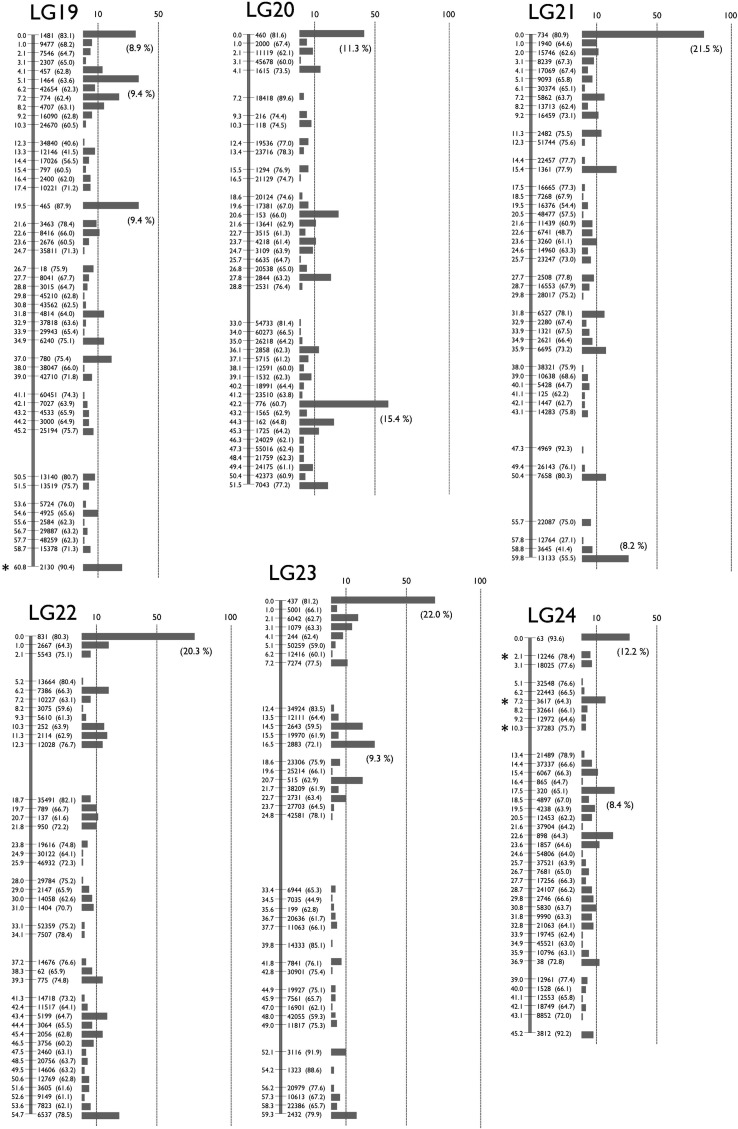
A Genetic linkage map of *K. marmoratus*/*K. hermaphroditus* based on RAD sequencing. The map coalesced into 24 linkage groups (LGs). LGs were named in decreasing order of total number of markers. For each LG, numbers to the left of the vertical bars represent map distances (cM, using the Kosambi function). Numbers to the right of the vertical bars give the names of representative markers with bootstrap values in the parenthesis (10,000 bootstrap replicates with AntMap). The horizontal bars at far right represent the total number of markers mapped to the same bin. The percentage of markers mapped to the same bins is shown in a parenthesis if ≥ 8.0% of the total markers on the LG. Markers showing segregation distortion are indicated by asterisks (*, *P* < 0.05; **, *P* < 0.01). Note that the concentration of markers is high at the tips of all LGs. Bins with the two highest numbers of markers, 119 and 118 markers, mapped to the middle of LG1 and the tip of LG5, respectively. Data are provided in File S1.

To make a genetic map, we mated *K. marmoratus* strain DAN and *K. hermaphroditus* strain PAN-RS (Figure 1 and Figure S1) by artificial fertilization (Nakamura *et al.* 2008). F_2_ were obtained by self-fertilization of a single F_1_ individual. Tags were generated by RAD-seq from genomic DNA of parental strains, F_1_, and 57 F_2_ progeny. For construction of a genotyping panel, we first selected 9937 polymorphic tags (markers) that are most likely unique in the genomes based on criteria described in the methods. The mapping panel was reduced to 49 F_2_ individuals by removing seven F_2_ fish with many missing genotypes and one individual with low heterozygosity. The genotyping panel was processed by genetic mapping software written in PHP (linkage.class.php in File S3). Many markers showed identical genotype patterns for the 49 F_2_ individuals and were thus not separated in the current cross, so the 9937 markers occupied 1157 bins of cosegregating markers. Then, recombination rates among all bins were calculated. Finally, bins were grouped by joining nearest neighbors judged by recombination rates (for further details, see MappingOutline.pdf in File S3). When 0.11–0.18 was used for maximum recombination rates between bin pairs, we obtained 24 major linkage groups (LGs), each of which had more than 20 bins. With a maximum recombination rate of 0.15, two minor groups containing two and three markers, and ten unlinked markers, were obtained in addition to 24 LGs. Twenty-four linkage groups corresponds to the known number of haploid chromosomes of both *K. marmoratus* and *K. hermaphroditus* (Scheel 1972; Sola *et al.* 1997; see also Figure S5). Next, by careful examination of recombination rates and consideration of possible genotyping errors, all markers but one from the two minor LGs and seven of the unlinked markers were successfully mapped to the 24 major LGs. Genotype correction involved 12 missing genotypes with near-threshold values for genotype calling parameters changed to homozygous genotypes because their minor alleles came from secondary reads of low quality. The remaining markers from a minor LG and three unlinked markers, together with 26 markers that initially mapped to the 24 LGs, were impossible to place unambiguously on the map: 14 markers with missing genotypes from more than four F_2_ individuals, 11 markers with generally low read depths, four markers that would introduce multiple double recombination events at adjacent bins, and one with over 200 read depth (average read depth was ∼60; segregation distorted to heterozygous with 0.0043 *P*-value and likely to represent a repetitive sequence). For each of 24 LGs, we further scrutinized markers with missing genotypes and markers that introduced double recombination events at adjacent bins. Because many of these markers contained genotypes with near-threshold values for genotype calling parameters, and their minor alleles came from secondary reads of low quality, we corrected genotypes of 90 markers from missing to homozygous, those of 26 markers from heterozygous to homozygous, and those of 9 markers from homozygous to missing genotypes. In several cases, manual bin reordering was necessary to minimize the number of recombination events (for example, markers 5515 and 1845 on LG8). As a result, a total of 1019 bins representing 9904 markers (99.7% of the initial 9937 markers) were mapped (Figure 2 and File S1). All mapped markers were heterozygous in the F_1_ with the exception of one marker that could not be genotyped. A total of 142 bins contained a single marker (1.4% of total mapped markers and 13.9% of total mapped bins, File S1). To minimize possible errors in bin orders, we rechecked genotypes of all the 142 bins (= markers) for recombination events and all of them had sufficient read coverage and high quality sequences. When those markers were eliminated from the analysis, the order of the remaining markers was identical. Genetic distances were calculated by the Kosambi function and the linkage groups were assigned numbers in order of the total number of markers (decreasing from 538/LG1 to 263/LG24, Table S2). Three tags (24452 on LG1, 59163 on LG19, and 29871 on LG24) were removed from the panel because their inclusion introduced double recombination events at adjacent bins on the map (see File S1). Because sequencing depth for these three markers was about twice most markers, we suspect that these markers originated not from a single locus, but from tandemly duplicated sequences. We confirmed our results by additional analyses with JoinMap 4.1 (Figure S2). The two mapping programs provided consistent marker grouping into LGs and marker ordering. For example, Figure S3 compares maps made by PHP and JoinMap. Because the two methods use slightly different algorithms, distances between markers sometimes differ. Next, markers with sequence conservation to platyfish were chosen as a subset of markers (see next section for details of sequence comparisons). For this limited set (440 out of 9904 markers), marker grouping into LGs was performed with a minimum LOD value of 6 and marker ordering was performed using the Maximum Likelihood algorithm in JoinMap 4.1. Maps made with this reduced set of markers with conserved sequence provided consistent marker grouping and marker order. For 18 LGs, markers grouped together with identical order (*e.g.*, LG1 in Figure S4), thus corroborating the order obtained using all markers. Exceptions involved LGs 4, 7, and 13, in which markers split into two groups each, and LGs 9, 20, and 21, for which most markers were in one group with a single marker not linked to those (see Figure S4). In addition, the final map (File S1) was shuffled and reanalyzed by the AntMap program, utilizing the “Ant Colony Optimization” method (Iwata and Ninomiya 2006) with the nearest neighboring locus strategy. With a recombination rate parameter between 0.09−0.24, a *P*-value parameter between 10^−6^ and 10^−11^, and a LOD score parameter up to 8.5, we again obtained the same 24 major linkage groups as with other programs. Bin order was calculated 10 times by the maximum log-likelihood method with 10,000 bootstrap replicates. Results showed a bin order identical to our final map (File S1). Thus, both bin grouping and locus ordering of the present map (Figure 2) by our custom-made program were confirmed by two independent and established mapping programs, demonstrating the utility of this simple mapping program.

Most bins (96.1%) segregated in Mendelian fashion but significant segregation distortion (*P* < 0.01) was found in two locations (Figure 2): LG5 (0 cM, distorted to heterozygous) and LG7 (15.4 cM, distorted to DAN homozygous). Additionally, stretches of markers with less distortion (*P* < 0.05) were present on LG4, LG7, and LG8. We excluded markers with significant segregation distortion and recalculated bin orders. Non-distorted markers again grouped together and in the same order as when including distorted markers, but with a large gap where distorted markers belong (data not shown). Because markers showing segregation distortion were analyzed independently of each other and yet cluster together in just a few specific map locations, segregation distortion likely results from a biological factor, probably due to some genomic incompatibility between the two species used in the cross.

The cumulative number of recombination events per chromosome ranged from 60–42, corresponding to 61.8–43.2 cM (1247.6 cM in total, Table S2). These values (an average of 52.0 cM/LG) indicate that approximately one recombination event occurred per meiosis (per homologous chromosome pair or bivalent), about a 50% probability of a single recombination event for both paternal and maternal chromosomes. In fact, the number of recombination events for each LG, shown in the last rows for each LG in File S1, were mostly 2, 1, or 0 in a 1:2:1 ratio (Table S2); no significant distortion was observed by chi square test (Table S2) except for LG2 (*P* < 0.05). This finding also supports the conclusion that approximately one recombination event occurred per chromosome per meiosis. On seven LGs (chromosomes), one or two F_2_ fish inherited one chromosome with a double recombination event and the other with one (a total of three recombination events per homologous chromosome pair). On LG8, one F_2_ fish (#69) inherited both maternal and paternal chromosomes with a double recombination event. A total of 11 (0.9%) double recombination events appeared in the present data. An average of one recombination event per bivalent generally occurs in most teleosts, which usually have about 24 haploid chromosomes: 56.5 cM/chromosome for medaka, Naruse *et al.* (2000); 55.3 cM/chromosome for platyfish, Amores *et al.* (2014); 63.1 cM/chromosome for halibut, Palaiokostas *et al.* (2013a); 59.5 cM/chromosome for a cichlid, Recknagel *et al.* (2013); and 49.0 cM/chromosome for tilapia, Palaiokostas *et al.* (2013b).

Map utility is a function of both the number of mapped markers and the number of progeny. Because the *K. marmoratus*/*K. hermaphroditus* RAD-seq map produced a large number of markers mapped on just 49 F_2_, an average of 9.8 markers were mapped to each bin (Table S2). Marker density (number of mapped markers per bin), however, was not evenly distributed across all chromosomes (Figure 2): chromosome tips contained a greater density of tags per cM compared to the middle of chromosomes. Only three chromosomes, LG1, 9, and 20, had bins with the greatest marker density in the middle of the chromosome.

Cytogenetics helps to interpret the distribution of markers we observed in the *K. marmoratus*/*K. hermaphroditus* genetic map. Scheel (1972) reported that the karyotype of *K. marmoratus* (strain not described) had n = 24, with 26 total haploid chromosome arms, and the karyotype of *K. hermaphroditus* (formerly *Kryptolebias ocellatus*, PAN-RS and HY, see Figure 1 and Figure S1) as n = 24 with 27 arms. Sola *et al.* (1997) showed that the metaphase karyotype of *K. marmoratus*, collected from seven localities, had two submetacentric chromosomes (with one short and one long arm) with 22 remaining acrocentrics (no visible short arms), thus agreeing with Scheel (1972). The two submetacentric chromosomes were the 15th and 16th in cytogenetic size (in decreasing order), and the short arm of the former contained a nucleolus organizing region (NOR). In the present study, we checked metaphase chromosomes of a *K. hermaphroditus* strain HY (Figure S5), which consisted of 21 acrocentrics with one subtelocentric and two submetacentrics as a haploid complement, again, agreeing with Scheel (1972). Similar to *K. marmoratus* as reported by Sola *et al.* (1997), HY submetacentrics were middle-sized in 24 chromosomes and one of them contained a faintly stained NOR. Because centromeres have been known to repress recombination (Nakaseko *et al.* 1986; Nachman 2002; Lynn *et al.* 2004; Baryshnikova *et al.* 2013), possibly through RNAi functions and histone methyltransferase (Ellermeier *et al.* 2010), the accumulation of many markers at one end of each chromosome in our study may well be due to the location of centromeres at or near one end of most chromosomes. LG1, 9, or 20 may correspond to one of the two submetacentric chromosomes that we and others have observed. Future fluorescent *in situ* hybridization (FISH) studies to assign genetic linkage groups to cytogenetic chromosomes could test this hypothesis. Additionally, the high density of markers (more than 8.0% of all tags on the LG) at both ends of nine chromosomes (LGs 2, 3, 4, 6, 7, 9, 11, 18, and 21) suggests that regions near their telomeres also showed reduced recombination. Brieuc *et al.* (2014) found similar recombination suppression around centromeres or telomeres in a Chinook salmon RAD-tag map. In contrast, Phillips *et al.* (2006) reported elevated recombination rates near the telomeres of zebrafish.

The mean map distance between adjacent bins ranged from 1.11–1.37 cM (average 1.22 cM) depending on the chromosome (Table S2). The map’s largest gap was only about 8 cM located on LG23; additional gaps occurred of 6 cM on LG 22 and 5 cM on LG 19, 21, and 23. These gaps may represent recombination hotspots (see Petes 2001; Lynn *et al.* 2004). Figure S6 depicts present map data as recombination density per marker so that putative recombination hotspots can be visualized easily. However, firm conclusions must wait for the comparison of physical maps to our genetic map.

### Conserved synteny of mangrove killifish, medaka, and platyfish genomes

**Figure 3:**
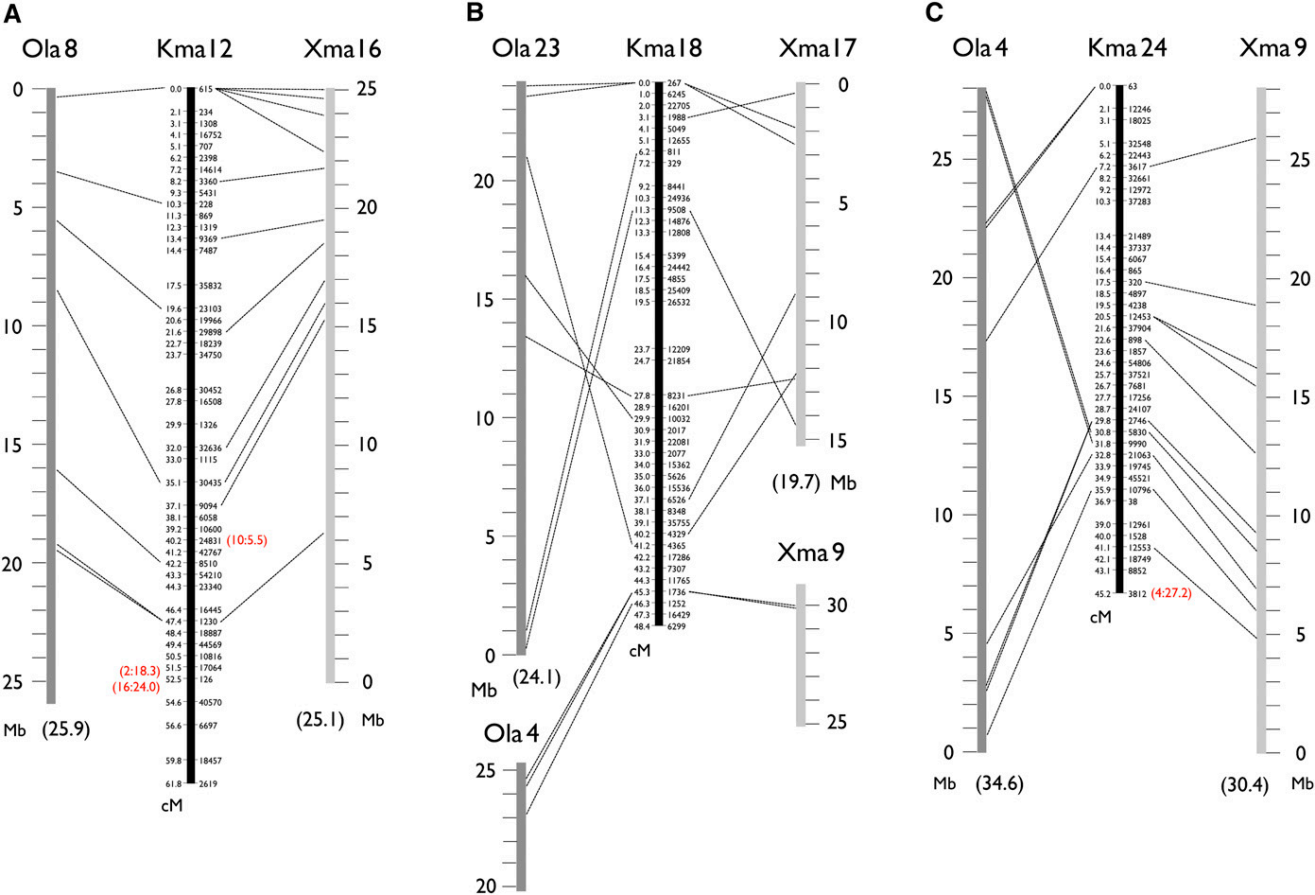
Representative conserved synteny of three *K marmoratus*/*K. hermaphroditus* linkage groups (Kma 12, 18, and 24) to platyfish (*X. maculatus*, Xma) and medaka (*O. latipes*, Ola) chromosomes suggesting, in general, a one-to-one relationship of *K. marmoratus*/*K. hermaphroditus* chromosomes to those of platyfish and medaka. Dotted lines indicate homology of mapped *K. marmoratus*/*K. hermaphroditus* markers to either Xma or Ola mapped genome sequences identified by the blastn program with default parameters. For platyfish, blast hits with a cut-off e-value of 1.0E-9 are shown. Kma 12 generally shares conserved syntenies to Xma 16 and Ola 8, including conserved order of sequences along the chromosomes (A). Some *K. marmoratus*/*K. hermaphroditus* markers show homology to sequences on non-orthologous chromosomes (indicated by red letters in parentheses). For example, a marker at 40.2 cM on Kma 12 has a homology to a sequence on Xma 10 at 5.5 Mb (A). Many *K. marmoratus*/*K. hermaphroditus* LGs show intrachromosomal rearrangements with respect to the other fish, probably due to inversions incurred after divergence from the last common ancestors of *K. marmoratus*/*K. hermaphroditus* and either platyfish or medaka (B). Results suggested a possible translocation involving Kma 18, which corresponds to Ola 23 or Xma 17 with a part of Ola 4 and Xma 9 attached, (B) and (C). Figure S7 provides data for all *K. marmoratus*/*K. hermaphroditus* LGs.

Because a full genome sequence is not yet available for either *K. marmoratus* or *K. hermaphroditus*, the following conserved synteny analysis remains fragmentary. We compared 9904 mapped markers (sequences available in File S2) to genomic sequences of platyfish (*Xiphophorus maculatus*) and medaka (*Oryzias latipes*), the next most closely related species with sequenced genomes that are anchored to genetic maps (named ‘chromonomes’ in Braasch *et al.* 2015). Platyfish is in the Cyprinodontiformes along with *K. marmoratus* and *K. hermaphroditus*, and medaka belongs to the sister order Beloniformes (see Setiamarga *et al.* 2008). Both species have 24 haploid chromosomes, as do *K. marmoratus* and *K. hermaphroditus* (Arai 2011), but the relationships of *K. marmoratus*/*K. hermaphroditus* chromosomes to the other species are as yet unclear. Out of 9904 markers, 440 (4.4%) and 269 (2.7%) had homology to similar sequences of platyfish and medaka, respectively; greater conservation is expected with platyfish because of historical relationships among species. A relatively low rate of conservation (4.4 and 2.7%) is natural because the majority of RAD-tags are derived from intergenic non protein-coding sequences. Conserved synteny analyses must compare “orthologs,” but it is difficult to make an unequivocal orthology table without a full genome sequence for *K. marmoratus*/*K. hermaphroditus*. Therefore, we first selected markers with a single hit (371 for platyfish and 176 for medaka). These hits had e-values not more than 6.0E-6. Blast hits with platyfish having e-values more than 1.0E-9 (50 markers) were further removed because they are more likely to be paralogous. Most markers on a single *K. marmoratus*/*K. hermaphroditus* linkage group showed homology to sequences from a single platyfish or a single medaka chromosome (Figure 3 and Figure S7). This result showed that each *K. marmoratus*/*K. hermaphroditus* chromosome corresponds in general to a single chromosome of either platyfish or medaka (thus making 24 orthologous chromosome pairs in all three species). For example, *K. marmoratus*/*K. hermaphroditus* (Kma) LG12 shares conserved syntenies to platyfish (Xma) LG16 and medaka (Ola) LG8, with both synteny and gene order conserved along chromosomes (Figure 3A). Sporadic translocations of relatively short segments, genome assembly errors, or errors in orthology assignments could explain many presumed “orthologs” scattered among non-orthologous chromosome pairs [20 (6.2%) in 321 “orthologs” for platyfish and *K. marmoratus*/*K. hermaphroditus* and 13 (7.4%) in 176 “orthologs” for medaka and *K. marmoratus*/*K. hermaphroditus*]. These markers are shown with red letters in parentheses in Figure 3 and Figure S7. Two such markers, 5064 (represented by 1948 on Kma LG1) and 34956 (represented by 1052 on Kma LG2), had homologous sequences on other orthologous chromosomes (Ola23-Kma18-Xma17 and Ola19-Kma17-Xma10, respectively), indicating that translocations happened after the *K. marmoratus*/*K. hermaphroditus* lineage diverged from the platyfish lineage (Figure S7). Similarly, markers 18609 (represented by 5464 on Kma LG2) and 38898 (represented by 2302 on Kma LG8), had homologous sequences on orthologous platyfish chromosomes (Xma18 and Xma1, respectively) but on non-orthologous medaka chromosomes (Ola22 and Ola19, respectively), indicating that translocations happened after the medaka lineage diverged from the Cyprinodontiformes. Nineteen out of 24 *K. marmoratus*/*K. hermaphroditus* chromosomes showed intrachromosomal rearrangements with respect to platyfish and medaka, probably due to inversions in one or more lineages. For example, Xma 18 (orthologous to Kma 2) may have experienced one inversion after the platyfish lineage diverged from the *K. marmoratus*/*K. hermaphroditus* lineage (Figure S7). Conversely, Kma 3 may have experienced one inversion after the *K. marmoratus*/*K. hermaphroditus* lineage diverged from the platyfish lineage. Several inversions were shared between platyfish and *K. marmoratus*/*K. hermaphroditus* with respect to medaka (Kma 4, 5, 6, 9, 14, and 24); these rearrangements may have occurred after the madaka lineage diverged from the Cyprinodontiformes. In general, however, large scale translocations between chromosomes seem to be rare; the only exception is Kma 18, which may be a fusion of Xma 17 with a part of Xma 9 (Ola 23 with a part of Ola 4) (Figure 3B). The remaining portion of Xma 9 or Ola 4 corresponds to Kma 24 (Figure 3C). This putative translocation occurred after the *K. marmoratus*/*K. hermaphroditus* lineage diverged from the platyfish lineage. The pattern of conserved synteny discussed above is similar to that observed between platyfish and medaka chromosomes, where genomic sequences were compared extensively (Amores *et al.* 2014) with many intrachromosomal but few interchromosomal rearrangements. At least among *K. marmoratus*/*K. hermaphroditus*, platyfish, and medaka, chromosomes tended to remain intact from the last common ancestor more than 100 million years ago (Steinke *et al.* 2006; Setiamarga *et al.* 2009) and translocations were few. However, the present analysis, based on just a few hundred conserved markers, may well have missed many genomic rearrangements. The anchoring of future genomic sequencing data to the present genetic map will make finer conserved synteny analysis possible. Although, in some cases, genomic scaffolds might not be resolved in the map relative to each other, they can be assigned to specific chromosome locations and the order could be optimized based on conserved synteny (Amores *et al.* 2014).

### Conclusions

In conclusion, we successfully constructed a RAD-seq based genetic linkage map for *K. marmoratus*/*K. hermaphroditus* that will be useful 1) to anchor rather short contigs and scaffolds assembled from next-generation sequencing; 2) for positional cloning of candidate genes from mutagenesis screening (Sucar *et al*. 2016); and 3) for mapping various QTL loci from wild fish or recombinant inbred lines, which can be obtained easily due to self-fertilization (Nakamura *et al.* 2008). The map will be an important resource for understanding the biology of hermaphroditism.

## 

## Supplementary Material

Supplemental Material
