# Supplementary material for: A Genetic Map for the Only Self-Fertilizing Vertebrate
Source: G3 (Bethesda). 2016 Feb 9;6(4):1095–106. doi: 10.1534/g3.115.022699 (PMC4825644; doi:10.1534/g3.115.022699)
Supplement: Supplemental Material [file supp_6_4_1095__index.html]

A Genetic Map for the Only Self-Fertilizing Vertebrate — Supplemental Material 

# A Genetic Map for the Only Self-Fertilizing Vertebrate

## Supplemental Material for Kanamori *et al.*, 2016

**Files in this Data Supplement:**

- Supporting Materials - File contains all Supporting Figures and Tables, and also contains legends for Supporting Files. (.pdf, 4,144 KB)
- Table S2 - Summary of the genetic map. (.pdf, 324 KB)
- Figure S1 - Phylogenetic relationship of hermaphroditic *Kryptolebias marmoratus* and *K. hermaphroditus* strains and a sister gonochoristic species, *K. caudomargimatus* with an outgroup, *K. brasiliensis*. (.pdf, 327 KB)
- Figure S2 - A Genetic linkage map of *Kryptplebias marmoratus/K. hermaphroditus* constructed with JoinMap 4.1. (.pdf, 1,542 KB)
- Figure S3 - A comparison of LG1 linkage maps of *Kryptplebias marmoratus/K. hermaphroditus* constructed with either JoinMap 4.1 or the PHP programs. (.pdf, 561 KB)
- Figure S4 - A comparison of genetic maps of *Kryptplebias marmoratus/K. hermaphroditus* constructed with a whole set of markers (whole) and with a subset of markers whose sequences showed conservations with platyfish (conserved). (.pdf, 873 KB)
- Figure S5 - Metaphase chromosomes of *Kryptolebias hermaphroditus* obtained from primary cultured fibroblasts of a HY strain adult. (.pdf, 305 KB)
- Figure S6 - A Genetic linkage map of *Kryptplebias marmoratus/K. hermaphroditus* based on RAD sequencing. (.pdf, 429 KB)
- Figure S7 - Conserved synteny of *Kryptolebias marmoratus/K. hermaphroditus* linkage groups (Kma LGs) to either platyfish (*Xiphophorus maculatus*, Xma) or medaka (*Oryzias latipes*, Ola) chromosomes, suggesting basically one to one relationship (24 orthologous chromosome pairs). (.pdf, 1,244 KB)
- Table S1 - Number of tags classified by presence (1) or absence (0) in each sample. (.pdf, 240 KB)
- File S1 - A genotyped panel of the map based on 9,904 markers and 49 F2 DNA (a Microsoft excel file). (.xlsx, 407 KB)
- File S2 - Nucleotide sequences of the markers used for mapping (a Microsoft excel file). (.xlsx, 540 KB)
- File S3 - A program used for linkage grouping and bin ordering. (.zip, 346 KB)
