# Supplementary material for: A Genetic Map for the Only Self-Fertilizing Vertebrate
Source: G3 (Bethesda). 2016 Feb 9;6(4):1095–106. doi: 10.1534/g3.115.022699 (PMC4825644; doi:10.1534/g3.115.022699)
Supplement: Supplemental Material [file supp_g3.115.022699_022699SM.pdf]

# A GENETIC MAP FOR THE ONLY SELF-FERTILIZING VERTEBRATE

Akira Kanamori[*, 1], Yosuke Sugita[*], Yasufumi Yuasa[*], Takamasa Suzuki[§, †], Kouichi Kawamura[‡], Yoshinobu Uno[**], Katsuyasu Kamimura[*], Yoichi Matsuda[**], Catherine A. Wilson[§§], Angel Amores[§§], John H. Postlethwait[§§], Koushirou Suga[††], and Yoshitaka Sakakura[††, 2]

[*] A Division of Biological Science, Graduate School of Science, [†]Japan Science and Technology Agency (JST) Exploratory Research for Advanced Technology (ERATO) Higashiyama Live-Holonics Project, and [**] Department of Applied Molecular Biosciences, Graduate School of Bioagricultural Sciences, Nagoya University, Nagoya, Aichi 464-8602, Japan, [§] Department of Biological Chemistry, College of Bioscience and Biotechnology, Chubu University, Kasugai, Aichi 487-8501, Japan, [‡]Graduate School of Bioresources, Mie University, Tsu, Mie 514-8507, Japan, [§§] Institute of Neuroscience, University of Oregon, Eugene, OR 97403-1254, and [††] Graduate School of Fisheries Science and Environmental Studies, Nagasaki University, Nagasaki 852-8521, Japan.

[1] Corresponding author: Division of Biological Science, Graduate School of Science, Nagoya University, Furocho, Chikusa, Nagoya 464-8602, Japan. E-mail: kanamori@bio.nagoya-u.ac.jp

[2] Corresponding author: Graduate School of Fisheries Science and Environmental Studies, Nagasaki University, 1-14 Bunkyo-machi, Nagasaki 852-8521, Japan. E-mail: sakakura@nagasaki-u.ac.jp

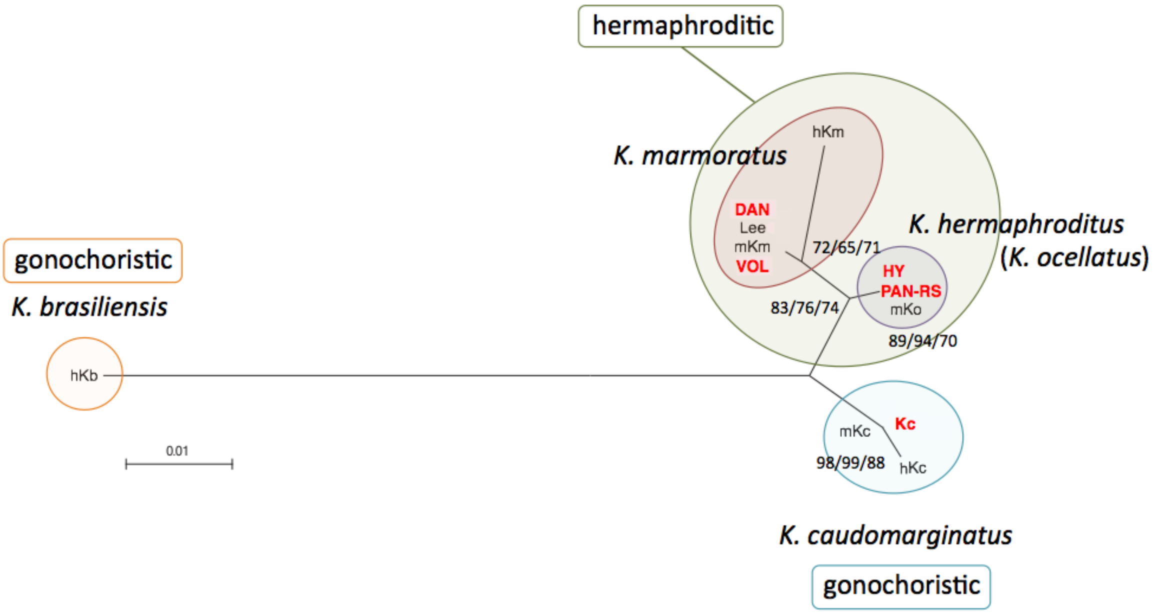


**Figure S1.** Phylogenetic relationship of hermaphroditic *Kryptolebias marmoratus* and *K. hermaphroditus* strains and a sister gonochoristic species, *K. caudomargimatus* with an outgroup, *K. brasiliensis*. The tree was constructed by neighbor-joining (NJ) method based on mitochondrial DNA sequences (a part of 12S rRNA, corresponding to nucleotide 17 to 332 in KP998194). Tree topology did not change when analyzed with maximum likelihood (ML) or maximum parsimony (MP) methods (data not shown). Numbers at the nodes (NJ/ML/MP) represent percentage recovery of those nodes per 10,000 bootstrap replicates. Only values above 60% are shown. The data from the present study are indicated in bold red. The hermaphroditic mangrove killifish is clearly separated into two groups, *K. marmoratus* and *K. hermaphroditus* (formerly *K. ocellatus*). The accession numbers are: hKb (AY946281) and hKm (AY946280) (Vermeulen and Hrbek, 2005); hKc (AF092294, Hrbek and Larson, 1999); mKc (AF002428), mKm (AF002429), and mKo (AF002430) (Murphy *et al*. 1999); Lee, Km (AF283503, Lee *et al*. 2001). Scale bars indicate genetic distance.

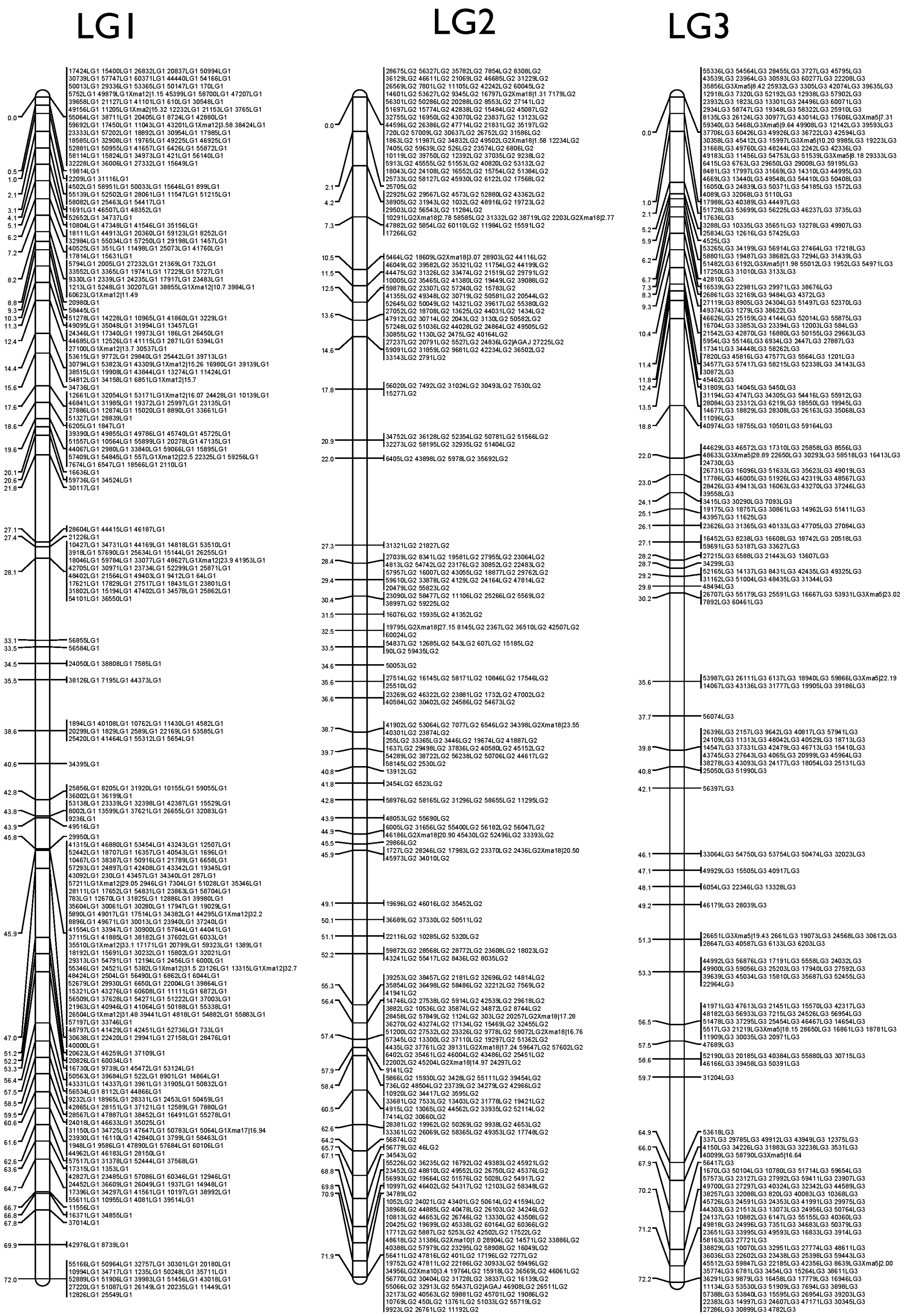
LG1
LG2
LG3

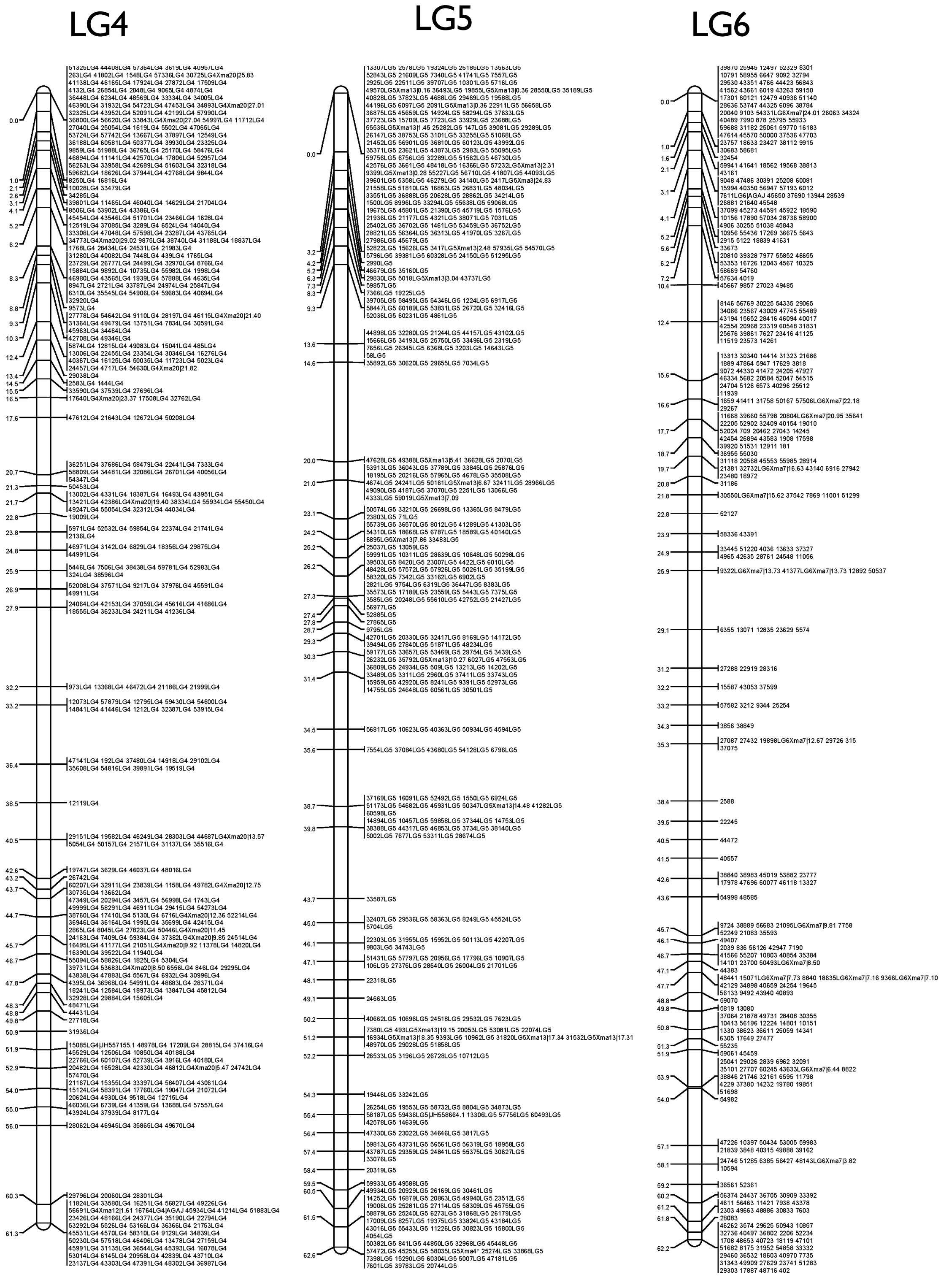
LG4
LG5
LG6

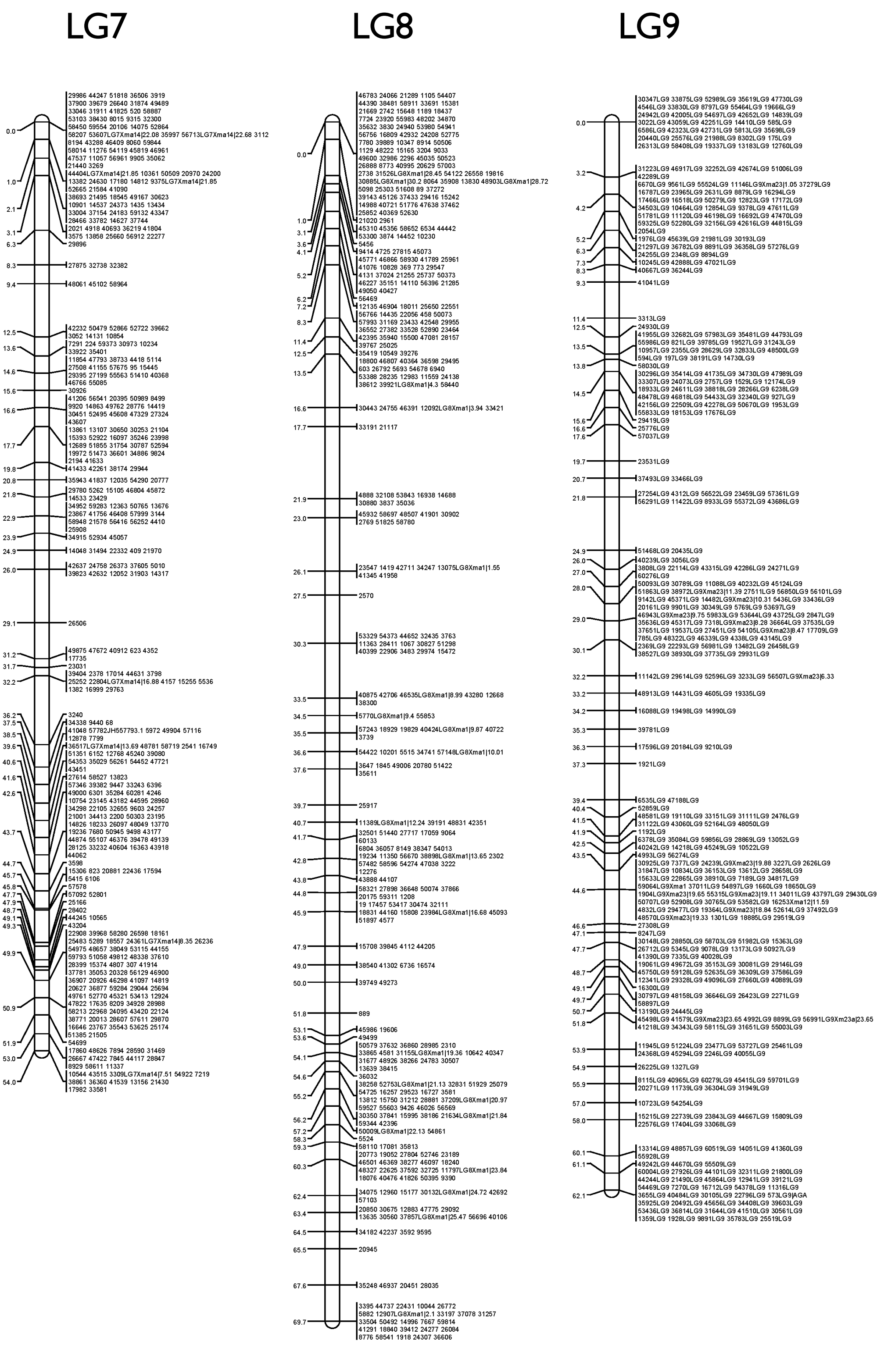
LG7
LG8
LG9

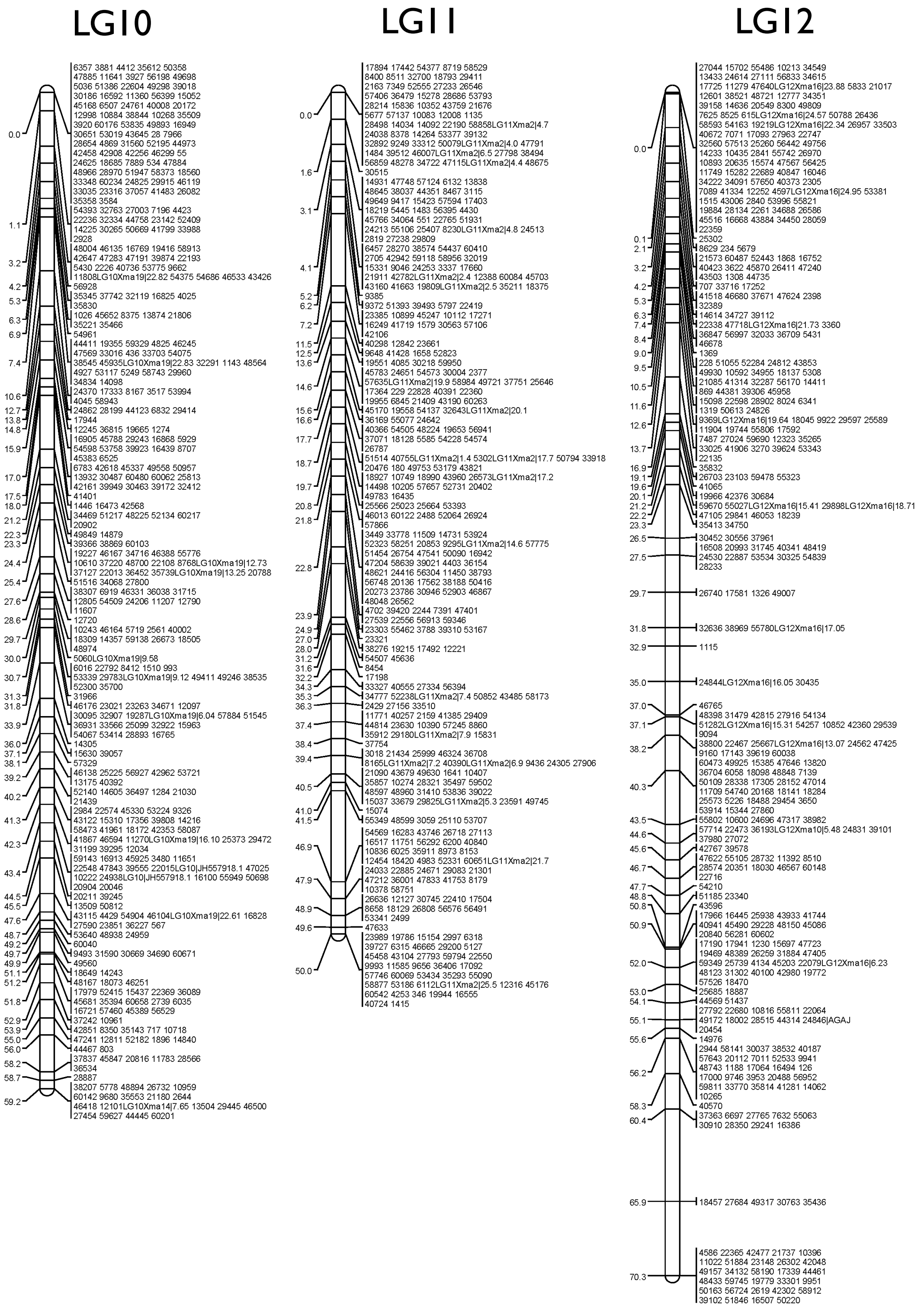
LG10
LG11
LG12

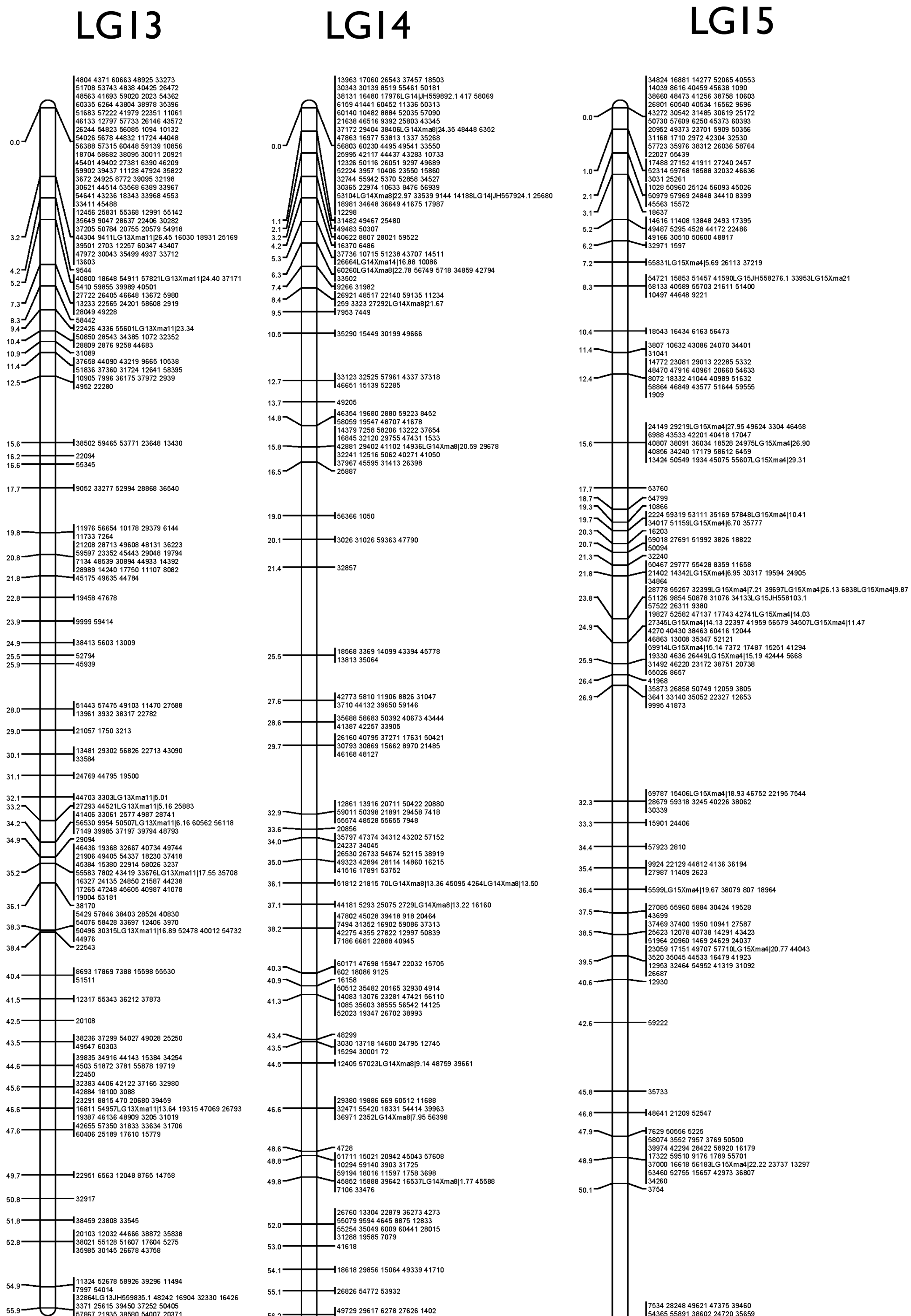
LG13
LG14
LG15

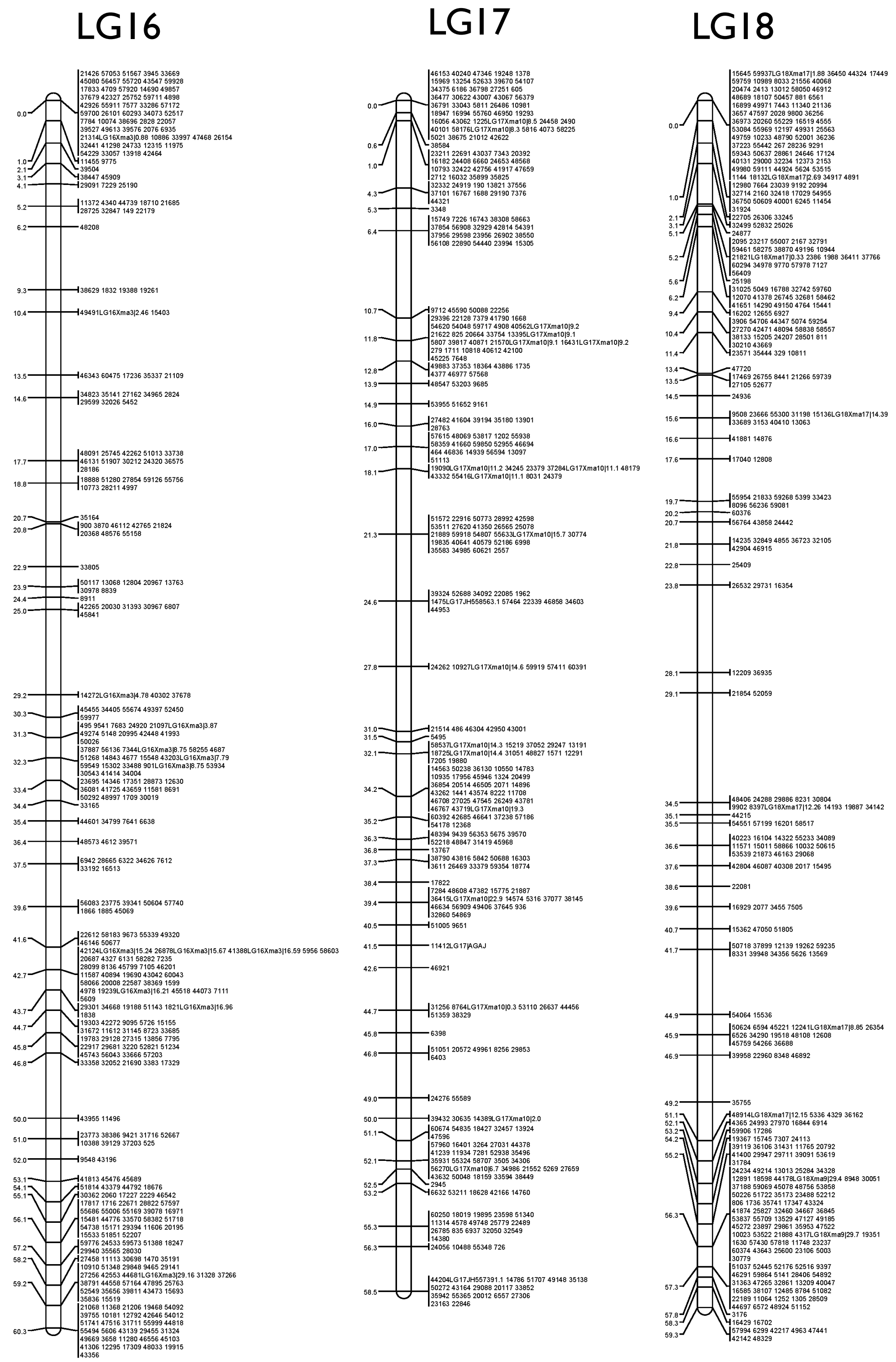
LG16
LG17
LG18

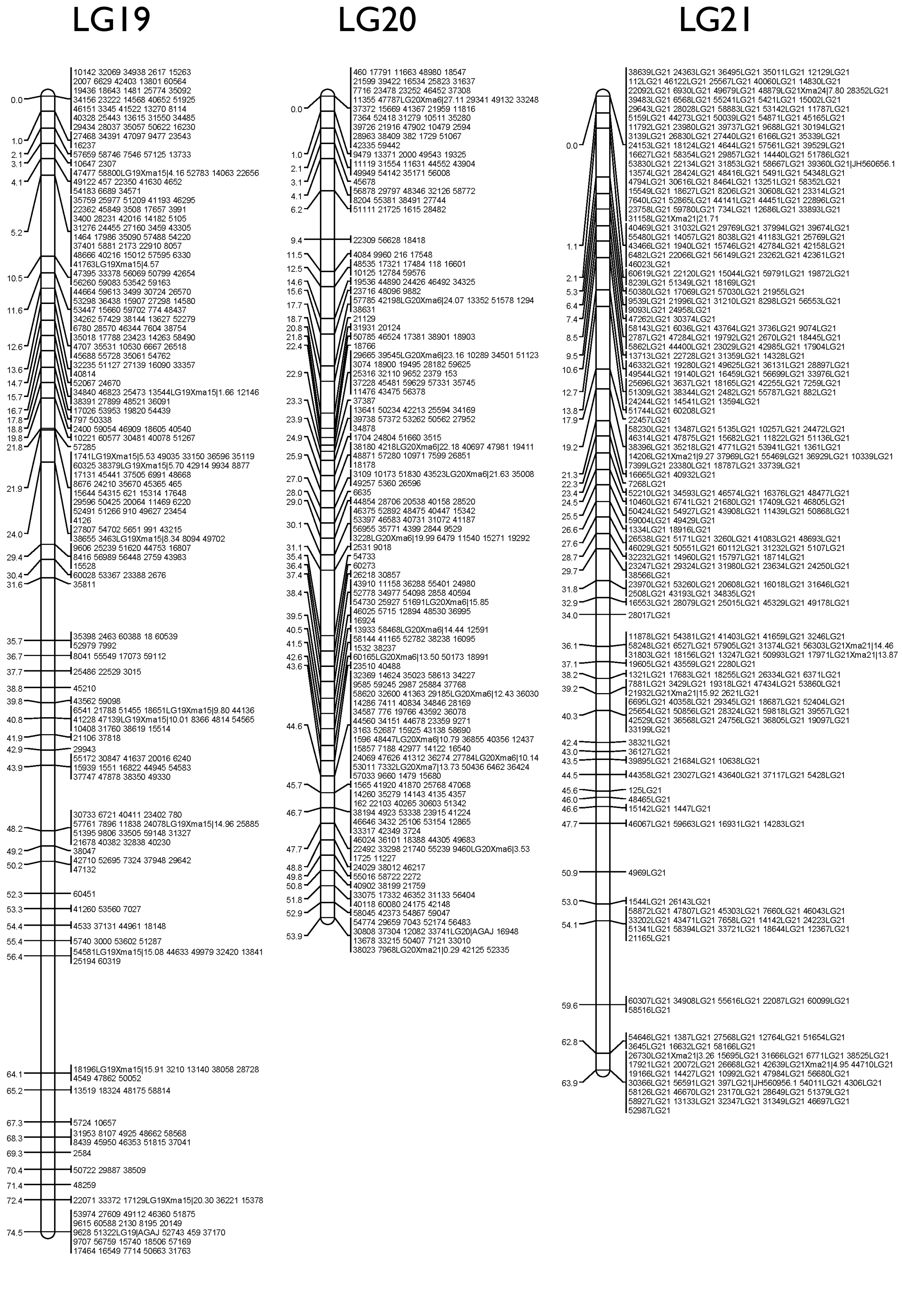
LG19
LG20
LG21

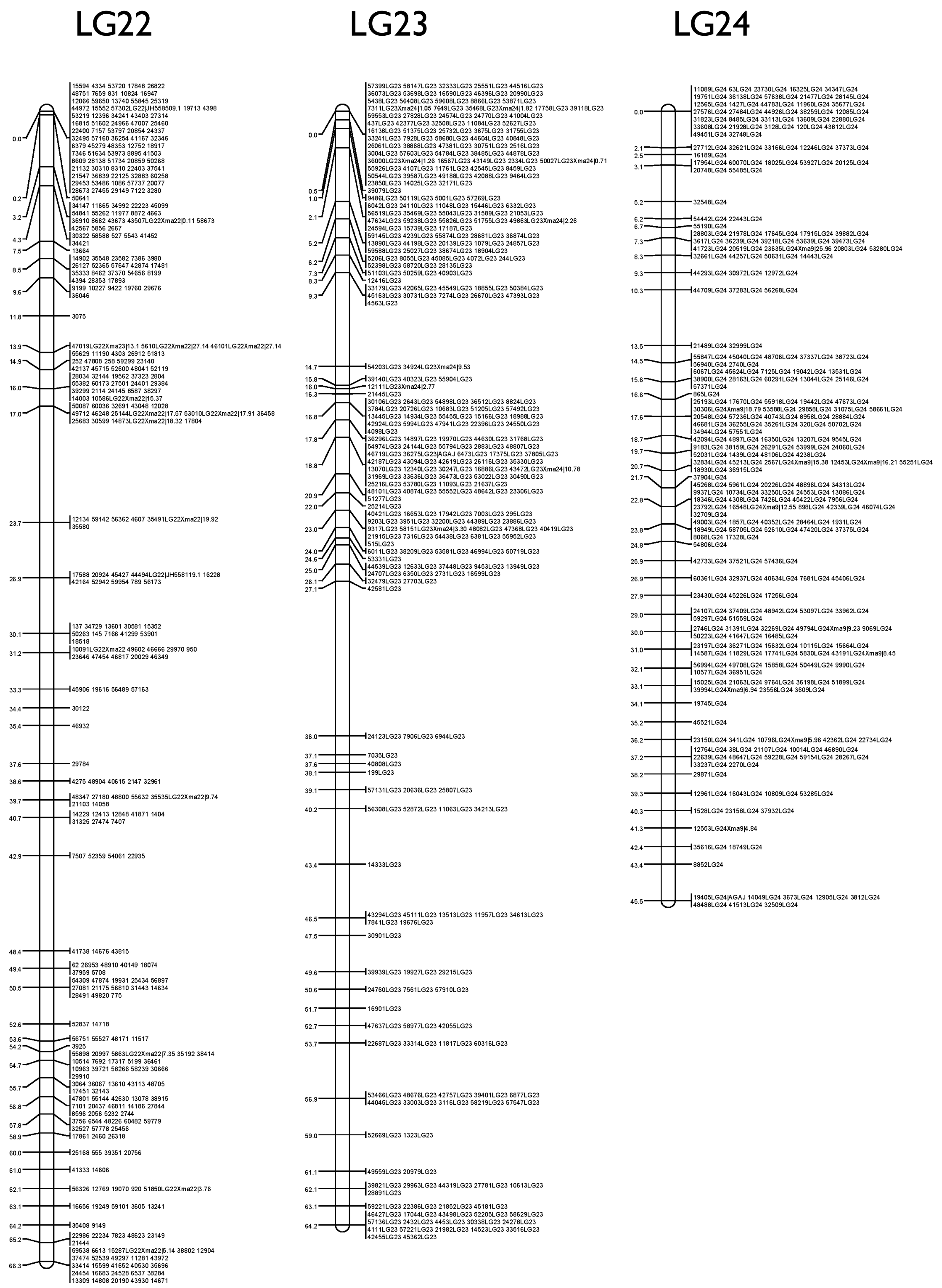
LG22
LG23
LG24

**Figure S2.** A Genetic linkage map of *Kryptplebias marmoratus*/*K. hermaphroditus* constructed with JoinMap 4.1. We obtained identical marker grouping into LGs as the PHP program and marker ordering consistent with the PHP program as shown in Figure 2.

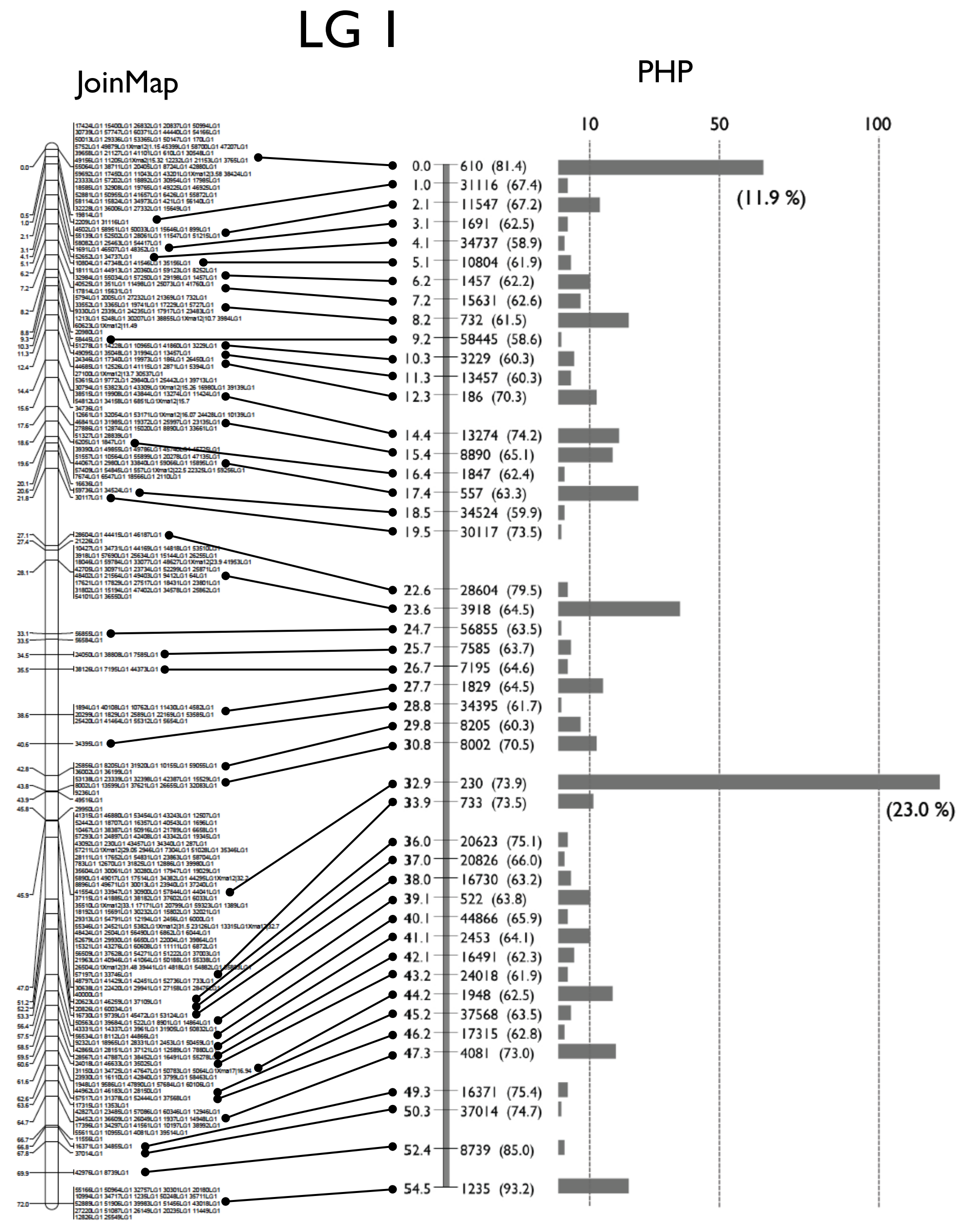


**Figure S3.** A comparison of LG1 linkage maps of *Kryptplebias marmoratus*/*K. hermaphroditus* constructed with either JoinMap 4.1 or the PHP programs. The marker grouping and locus ordering are consistent in both programs. However, the distances between markers were calculated differently in some cases because the algorithms used in two programs were distinct.

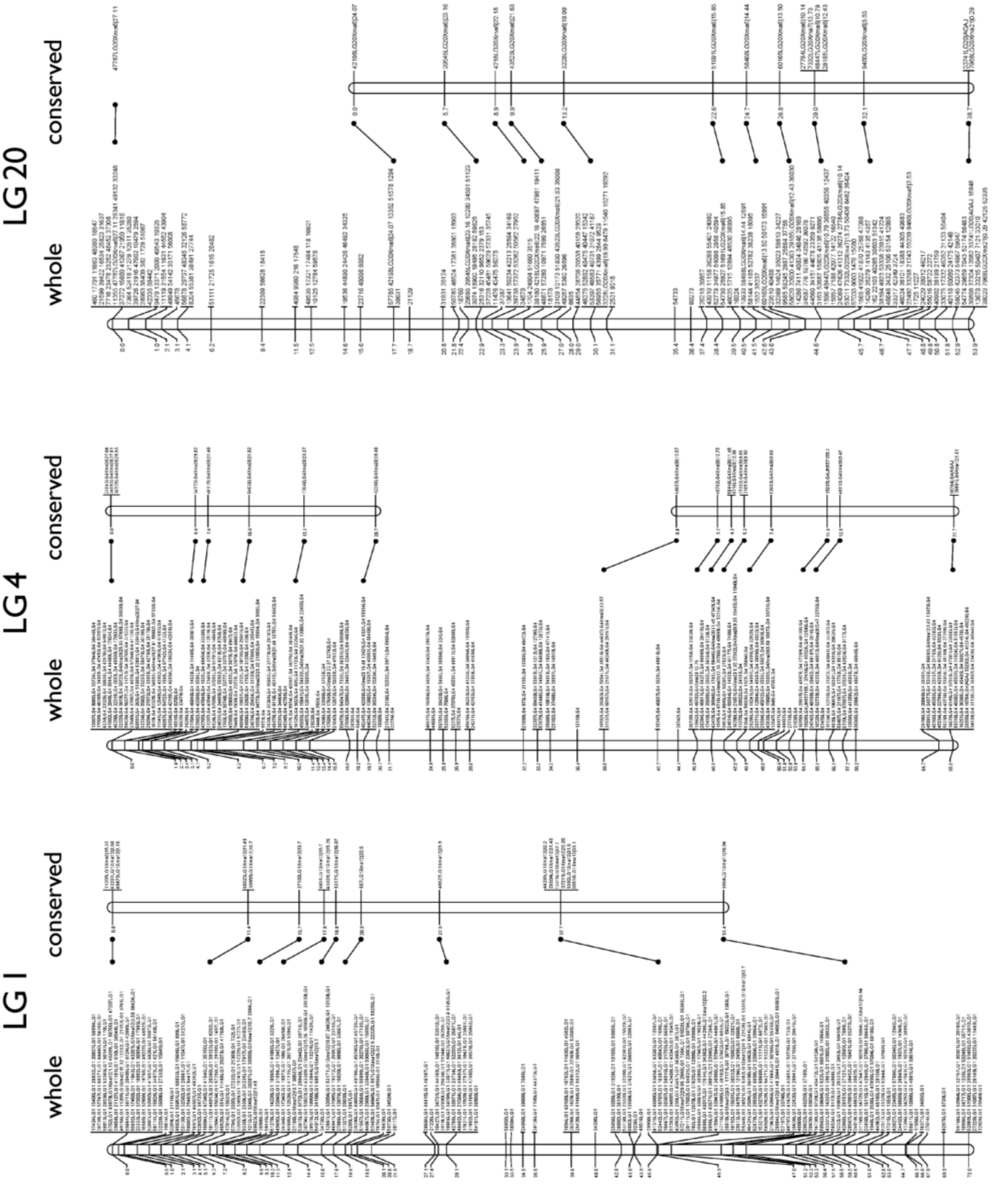


**Figure S4.** A comparison of genetic maps of *Kryptplebias marmoratus*/*K. hermaphroditus* constructed with a whole set of markers (whole) and with a subset of markers whose sequences showed conservations with platyfish (conserved). Marker grouping and marker ordering are consistent in both maps. For 18 LGs, the markers were grouped together and were ordered identically (e.g. LG1). The results corroborate the ordering obtained in the whole map. The exceptions were LG4 (together with LGs 7 and 13) where the markers were in two groups or LG20 (together with LGs 9 and 21) where most markers were in one group with a single marker not linked to those.

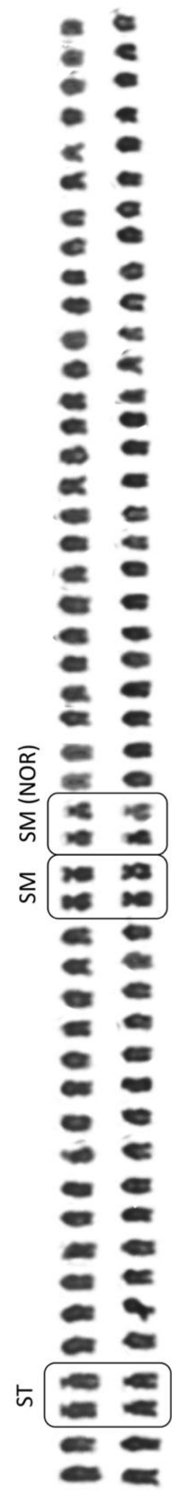


**Figure S5.** Metaphase chromosomes of *Kryptolebias hermaphroditus* obtained from primary cultured fibroblasts of a HY strain adult. Two sets of representative Giemsa stained samples shown were composed of 48 chromosomes. Total of 23 spreads were counted and 48 was a mode (data not shown). Except for one pair of subtelocentric (ST) and two pairs of submetacentric (SM) chromosomes, all were acrocentric chromosomes gradually changing in size. One pair of SM chromosomes had less stained areas in their short arms, probably representing a nucleolus organizing region (NOR).

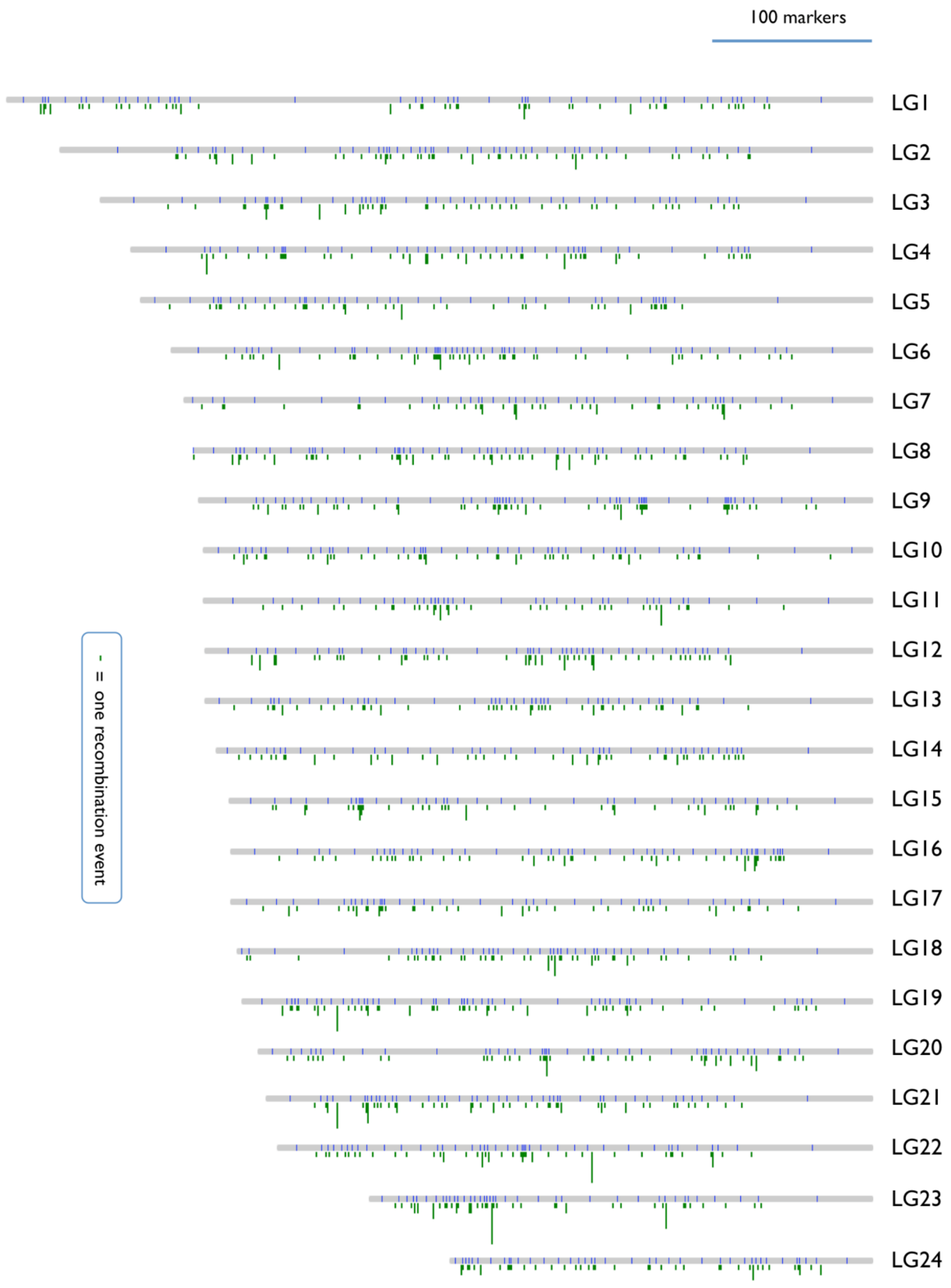
100 markers
LG1
LG2
LG3
LG4
LG5
LG6
LG7
LG8
LG9
LG10
LG11
LG12
LG13
LG14
LG15
LG16
LG17
LG18
LG19
LG20
LG21
LG22
LG23
LG24
= one recombination event

**Figure S6.** A Genetic linkage map of *Kryptplebias marmoratus*/*K. hermaphroditus* based on RAD sequencing. The map was coalesced into 24 linkage groups (LGs). LGs were named in decreasing order of total number of markers, relative to which each vertical bar (grey) was drawn. Each bin (location of co-segregating markers) occupies length relative to total number of co-segregating markers, middle of which blue horizontal lines were plotted. Green horizontal bars, drawn on the right of the grey bars, represent numbers of recombination events and were plotted between adjacent bins. Assuming relatively even distribution of markers, these grey bars represent physical maps with density of green bars as relative frequency of recombination events along the chromosomes.

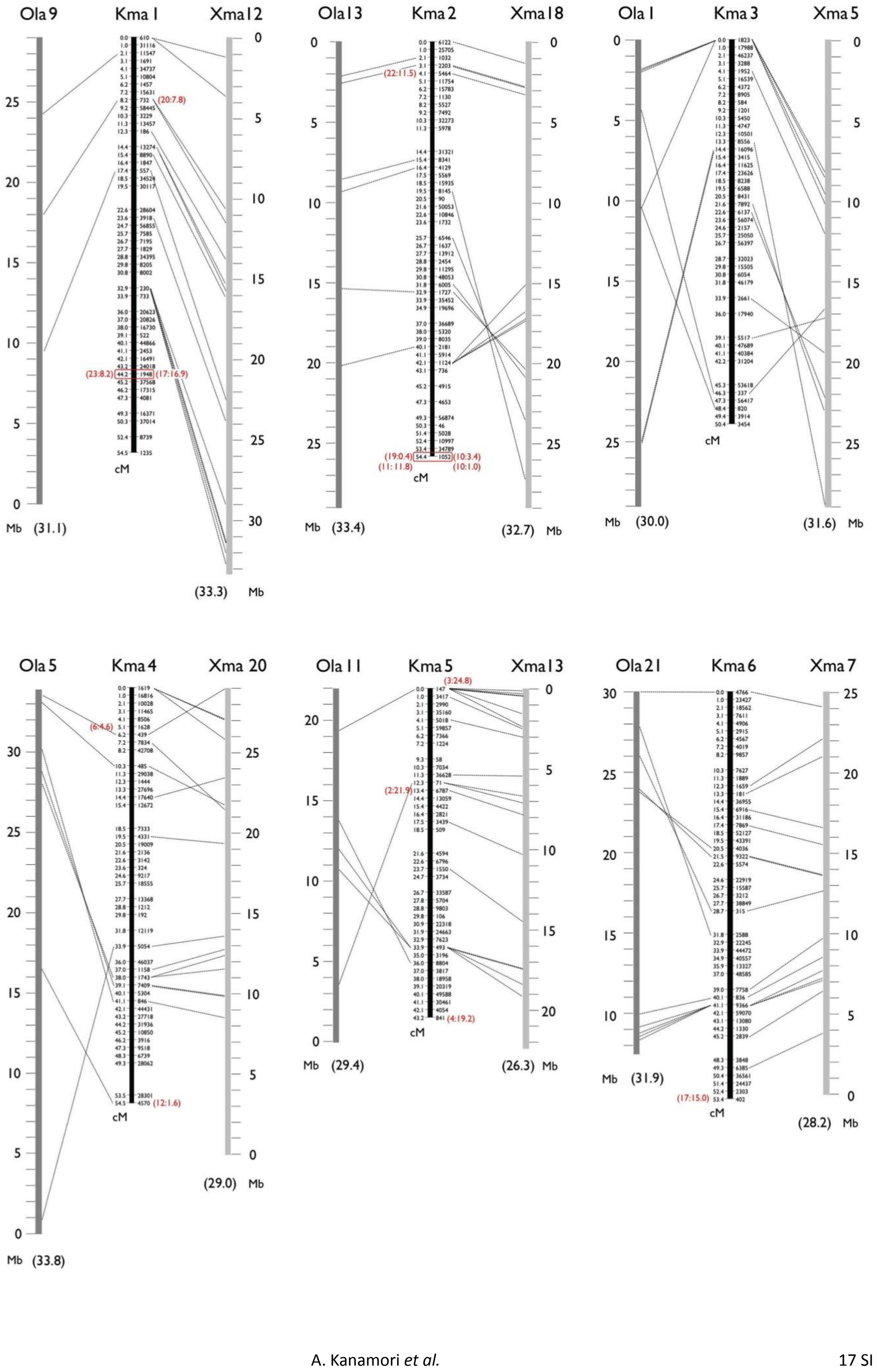
Ola 9
Kma 1
Xma 12
Ola 13
Kma 2
Xma 18
Ola 1
Kma 3
Xma 5
Ola 5
Kma 4
Xma 20
Ola 11
Kma 5
Xma 13
Ola 21
Kma 6
Xma 7
Mb (31.1)
(33.3) Mb
Mb (33.4)
(32.7) Mb
Mb (30.0)
(31.6) Mb
Mb (33.8)
(29.0) Mb
Mb (29.4)
(26.3) Mb
Mb (31.9)
(28.2) Mb
cM

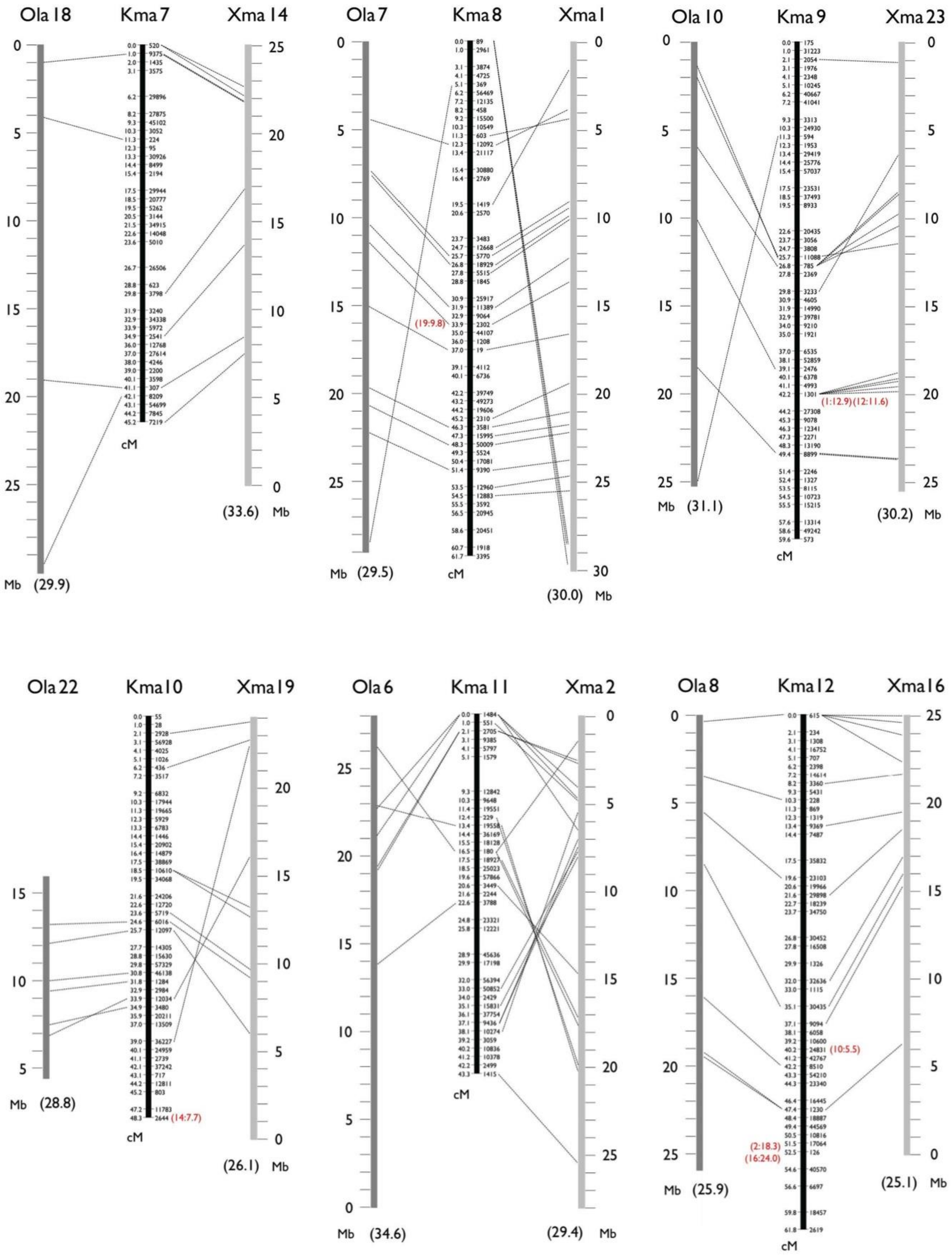

Ola 18
Kma 7
Xma 14
Ola 7
Kma 8
Xma 1
Ola 10
Kma 9
Xma 23
Mb (29.9)
(33.6) Mb
Mb (29.5)
(30.0) Mb
Mb (31.1)
(30.2) Mb
cM
Ola 22
Kma 10
Xma 19
Ola 6
Kma 11
Xma 2
Ola 8
Kma 12
Xma 16
Mb (28.8)
(26.1) Mb
Mb (34.6)
(29.4) Mb
Mb (25.9)
(25.1) Mb

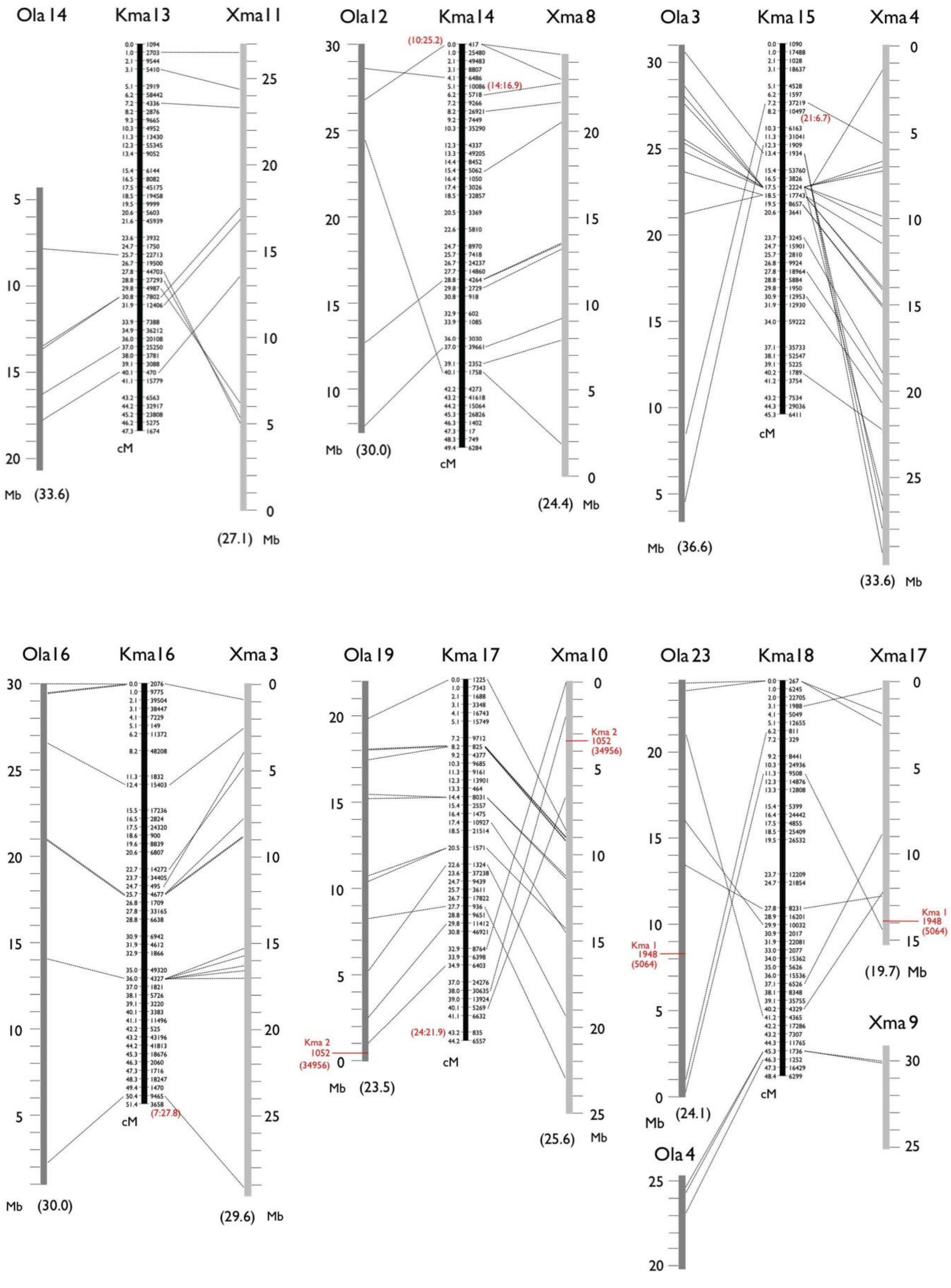
Ola14
Kma13
Xma11
Ola12
Kma14
Xma8
Ola3
Kma15
Xma4
Ola16
Kma16
Xma3
Ola19
Kma17
Xma10
Ola23
Kma18
Xma17
Xma9
Ola4

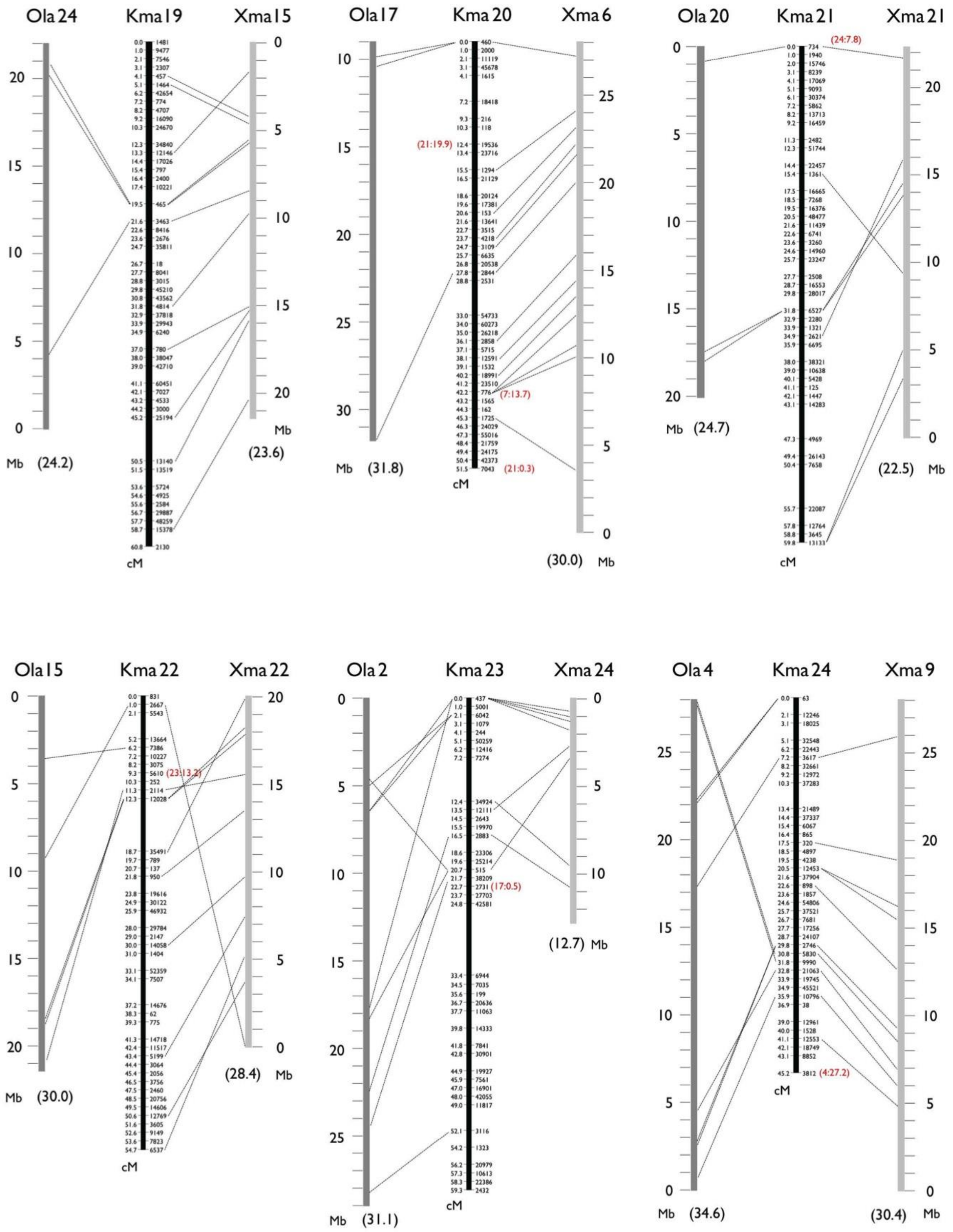
Ola 24
Kma 19
Xma 15
Mb (24.2)
(23.6)
cM
Ola 17
Kma 20
Xma 6
(21:19.9)
(7:13.7)
(21:0.3)
Mb (31.8)
(30.0) Mb
Ola 20
Kma 21
Xma 21
(24:7.8)
Mb (24.7)
(22.5) Mb
Ola 15
Kma 22
Xma 22
(23:13.2)
Mb (30.0)
(28.4) Mb
Ola 2
Kma 23
Xma 24
(17:0.5)
(12.7) Mb
Mb (31.1)
Ola 4
Kma 24
Xma 9
(4:27.2)
Mb (34.6)
(30.4) Mb

**Figure S7.** Conserved synteny of *Kryptolebias marmoratus*/*K. hermaphroditus* linkage groups (Kma LGs) to either platyfish (*Xiphophorus maculatus*, Xma) or medaka (*Oryzias latipes*, Ola) chromosomes, suggesting basically one to one relationship (24 orthologous chromosome pairs). Dotted lines indicate homology of mapped Kma markers to either Xma or Ola mapped genome sequences identified by the blastn program with default parameters. For platyfish, blast hits with a cut-off e-value of 1.0E-9 are shown. Many Kma LGs show intrachromosomal rearrangements probably due to recurred inversions after divergence from common ancestors between either Xma or Ola. Some Kma markers show homology to sequences on non-orthologous chromosomes (indicated by red letters in parenthesis). For example, a marker at 8.2 cM on Kma LG1 (Kma1) has a homology to a sequence on Xma chromosome 20 at 7.8 Mb. Two such markers, 5064 (represented by 1948 on Kma LG1) and 34956 (represented by 1052 on Kma LG2), had homologous sequences on another orthologous chromosomes (Ola23-Kma18-Xma17 and Ola19-Kma17-Xma10, respectively), indicating translocations happened after the *K. marmoratus*/*K. hermaphroditus* lineage diverged from platyfish. Similarly, markers 18609 (represented by 5464 on Kma LG2) and 38898 (represented by 2302 on Kma LG8), had homologous sequences on orthologous platy chromosomes (Xma18 and Xma1, respectively) but on non-orthologous medaka chromosomes (Ola22 and Ola19, respectively), indicating translocations happened after the medaka lineage diverged from cyprinodontiformes.

**File S1** A genotyped panel of the map based on 9,904 markers and 49 $F_2$ DNA (a Microsoft excel file). Twenty-four linkage groups (LGs) are separated and bins of co-segregating markers have been ordered. Genotype symbols represent following: a, DAN homozygous; b, PAN-RS homozygous; h, heterozygous; -, missing genotype.

**File S2** Nucleotide sequences of the markers used for mapping (a Microsoft excel file).

**File S3** A program used for linkage grouping and bin ordering. The original genotyping panel with 49 $F_2$ individuals and 9,937 markers (map49F2_42.txt), PHP scripts, and an outline of the program (MappingOutline.pdf) were provided. Please consult "Readme.txt" for running the scripts.

Files S1, S2, and S3 are available for download at www.g3journal.org/lookup/suppl/doi:10.1534/g3.115.022699/-/DC1

**Table S1. Number of tags classified by presence (1) or absence (0) in each sample.**

| | *K. marmoratus* | | *K.hermaphroditus* | | | |
|---|---|---|---|---|---|---|
| sample | DAN | VOL | PAN-RS | HY | Kc | number of tags |
| | 1 | 1 | 1 | 1 | 1 | 35415 |
| | 0 | 1 | 1 | 1 | 1 | 98 |
| | 1 | 0 | 1 | 1 | 1 | 116 |
| | 1 | 1 | 0 | 1 | 1 | 170 |
| | 1 | 1 | 1 | 0 | 1 | 212 |
| | 1 | 1 | 1 | 1 | 0 | 16426 |
| | 0 | 0 | 1 | 1 | 1 | 672 |
| | 0 | 1 | 0 | 1 | 1 | 8 |
| | 0 | 1 | 1 | 0 | 1 | 5 |
| | 0 | 1 | 1 | 1 | 0 | 132 |
| | 1 | 0 | 0 | 1 | 1 | 4 |
| | 1 | 0 | 1 | 0 | 1 | 7 |
| | 1 | 0 | 1 | 1 | 0 | 162 |
| | 1 | 1 | 0 | 0 | 1 | 489 |
| | 1 | 1 | 0 | 1 | 0 | 235 |
| | 1 | 1 | 1 | 0 | 0 | 279 |
| | 0 | 0 | 0 | 1 | 1 | 72 |
| | 0 | 0 | 1 | 0 | 1 | 49 |
| | 0 | 0 | 1 | 1 | 0 | 1752 |
| | 0 | 1 | 0 | 0 | 1 | 38 |
| | 0 | 1 | 0 | 1 | 0 | 33 |
| | 0 | 1 | 1 | 0 | 0 | 22 |
| | 1 | 0 | 0 | 0 | 1 | 22 |
| | 1 | 0 | 0 | 1 | 0 | 29 |
| | 1 | 0 | 1 | 0 | 0 | 45 |
| | 1 | 1 | 0 | 0 | 0 | 1975 |
| | 1 | 0 | 0 | 0 | 0 | 347 |
| | 0 | 1 | 0 | 0 | 0 | 391 |
| | 0 | 0 | 1 | 0 | 0 | 646 |
| | 0 | 0 | 0 | 1 | 0 | 717 |
| | 0 | 0 | 0 | 0 | 1 | 18679 |
| total | 57214 | 57217 | 57489 | 57361 | 56859 | 79247 |

identical 9240
polymorphic 26175
(total SNPs 53544)

**Table S2. Summary of the genetic map.**

| LG | total recombination events | cM (Kosambi) | total markers per LG | total bins per LG | gaps≥3 | average markers per bin | average map distance between adjacent bins (cM) | recombination events per LG | | | | | |
|---|---|---|---|---|---|---|---|---|---|---|---|---|---|
| | | | | | | | | 4 | 3 | 2 | 1 | 0 | P (chi square) |
| 1 | 53 | 54.5 | 538 | 46 | 3 | 11.70 | 1.18 | 0 | 1 | 14 | 22 | 12 | 0.775 |
| 2 | 53 | 54.4 | 505 | 47 | 3 | 10.74 | 1.16 | 0 | 0 | 19 | 15 | 15 | * 0.018 |
| 3 | 49 | 50.4 | 480 | 43 | 3(2) | 11.16 | 1.17 | 0 | 0 | 14 | 21 | 14 | 0.607 |
| 4 | 53 | 54.5 | 461 | 44 | 4, 3 | 10.48 | 1.24 | 0 | 0 | 13 | 27 | 9 | 0.559 |
| 5 | 42 | 43.2 | 455 | 39 | 3 | 11.67 | 1.11 | 0 | 0 | 9 | 24 | 16 | 0.364 |
| 6 | 52 | 53.4 | 436 | 46 | 3(2) | 9.48 | 1.16 | 0 | 0 | 15 | 22 | 12 | 0.645 |
| 7 | 44 | 45.2 | 428 | 37 | 3(2) | 11.57 | 1.22 | 0 | 0 | 10 | 24 | 15 | 0.594 |
| 8 | 60 | 61.7 | 422 | 49 | 3(2) | 8.61 | 1.26 | 1 | 2 | 16 | 18 | 12 | 0.237 |
| 9 | 58 | 59.6 | 419 | 50 | 3 | 8.38 | 1.19 | 0 | 0 | 19 | 20 | 10 | 0.084 |
| 10 | 47 | 48.3 | 416 | 43 | | 9.67 | 1.12 | 0 | 0 | 11 | 25 | 13 | 0.912 |
| 11 | 42 | 43.3 | 416 | 36 | 4, 3 | 11.56 | 1.20 | 0 | 0 | 6 | 30 | 13 | 0.107 |
| 12 | 60 | 61.8 | 415 | 45 | 3(3) | 9.22 | 1.37 | 0 | 0 | 17 | 26 | 6 | 0.077 |
| 13 | 46 | 47.3 | 415 | 42 | | 9.88 | 1.13 | 0 | 0 | 10 | 26 | 13 | 0.759 |
| 14 | 48 | 49.4 | 408 | 41 | | 9.95 | 1.20 | 0 | 1 | 12 | 21 | 15 | 0.571 |
| 15 | 44 | 45.3 | 400 | 36 | 3(2) | 11.11 | 1.26 | 0 | 0 | 8 | 28 | 13 | 0.364 |
| 16 | 50 | 51.4 | 399 | 43 | 3(2) | 9.28 | 1.20 | 0 | 1 | 12 | 23 | 13 | 0.931 |
| 17 | 43 | 44.2 | 399 | 38 | | 10.50 | 1.16 | 0 | 0 | 8 | 27 | 14 | 0.372 |
| 18 | 47 | 48.4 | 395 | 41 | 4, 3 | 9.63 | 1.18 | 0 | 0 | 15 | 17 | 17 | 0.093 |
| 19 | 59 | 60.8 | 392 | 48 | 5 | 8.17 | 1.27 | 0 | 0 | 15 | 29 | 5 | 0.057 |
| 20 | 50 | 51.5 | 382 | 42 | 4, 3 | 9.10 | 1.23 | 0 | 0 | 12 | 26 | 11 | 0.894 |
| 21 | 58 | 59.8 | 377 | 44 | 5, 4 | 8.57 | 1.36 | 0 | 1 | 17 | 21 | 10 | 0.252 |
| 22 | 53 | 54.7 | 370 | 41 | 6, 3(2) | 9.02 | 1.33 | 0 | 1 | 12 | 26 | 10 | 0.775 |
| 23 | 57 | 59.3 | 313 | 39 | 8, 5, 3 | 8.03 | 1.52 | 0 | 2 | 14 | 23 | 10 | 0.686 |
| 24 | 44 | 45.2 | 263 | 39 | 3 | 6.74 | 1.16 | 0 | 0 | 10 | 24 | 15 | 0.594 |
| sum | 1212 | 1247.6 | 9904 | 1019 | — | — | — | 1 | 9 | 308 | 565 | 293 | — |
| (average) | (50.5) | (52.0) | (412.7) | (42.5) | | (9.76) | (1.22) | | | | | | |
