# Supplementary material for: A Genetic Map for the Only Self-Fertilizing Vertebrate
Source: G3 (Bethesda). 2016 Feb 9;6(4):1095–106. doi: 10.1534/g3.115.022699 (PMC4825644; doi:10.1534/g3.115.022699)
Supplement: Supplemental Material [file supp_g3.115.022699_TableS2.pdf]

Table S2. Summary of the genetic map.

LG	total recombination events	cM (Kosambi)	total markers per LG	total bins per LG	gaps≥3	average markers per bin	average map distance between adjacent bins (cM)	recombination events per LG: 4	3	2	1	0	P (chi square)
1	53	54.5	538	46	3	11.70	1.18	0	1	14	22	12	0.775
2	53	54.4	505	47	3	10.74	1.16	0	0	19	15	15	* 0.018
3	49	50.4	480	43	3(2)	11.16	1.17	0	0	14	21	14	0.607
4	53	54.5	461	44	4, 3	10.48	1.24	0	0	13	27	9	0.559
5	42	43.2	455	39	3	11.67	1.11	0	0	9	24	16	0.364
6	52	53.4	436	46	3(2)	9.48	1.16	0	0	15	22	12	0.645
7	44	45.2	428	37	3(2)	11.57	1.22	0	0	10	24	15	0.594
8	60	61.7	422	49	3(2)	8.61	1.26	1	2	16	18	12	0.237
9	58	59.6	419	50	3	8.38	1.19	0	0	19	20	10	0.084
10	47	48.3	416	43		9.67	1.12	0	0	11	25	13	0.912
11	42	43.3	416	36	4, 3	11.56	1.20	0	0	6	30	13	0.107
12	60	61.8	415	45	3(3)	9.22	1.37	0	0	17	26	6	0.077
13	46	47.3	415	42		9.88	1.13	0	0	10	26	13	0.759
14	48	49.4	408	41		9.95	1.20	0	1	12	21	15	0.571
15	44	45.3	400	36	3(2)	11.11	1.26	0	0	8	28	13	0.364
16	50	51.4	399	43	3(2)	9.28	1.20	0	1	12	23	13	0.931
17	43	44.2	399	38		10.50	1.16	0	0	8	27	14	0.372
18	47	48.4	395	41	4, 3	9.63	1.18	0	0	15	17	17	0.093
19	59	60.8	392	48	5	8.17	1.27	0	0	15	29	5	0.057
20	50	51.5	382	42	4, 3	9.10	1.23	0	0	12	26	11	0.894
21	58	59.8	377	44	5, 4	8.57	1.36	0	1	17	21	10	0.252
22	53	54.7	370	41	6, 3(2)	9.02	1.33	0	1	12	26	10	0.775
23	57	59.3	313	39	8, 5, 3	8.03	1.52	0	2	14	23	10	0.686
24	44	45.2	263	39	3	6.74	1.16	0	0	10	24	15	0.594
sum	1212	1247.6	9904	1019	—	—	—	1	9	308	565	293	—
(average)	(50.5)	(52.0)	(412.7)	(42.5)		(9.76)	(1.22)						
