# Supplementary material for: A Genetic Map for the Only Self-Fertilizing Vertebrate
Source: G3 (Bethesda). 2016 Feb 9;6(4):1095–106. doi: 10.1534/g3.115.022699 (PMC4825644; doi:10.1534/g3.115.022699)
Supplement: Supplemental Material [file supp_g3.115.022699_FigureS1.pdf]

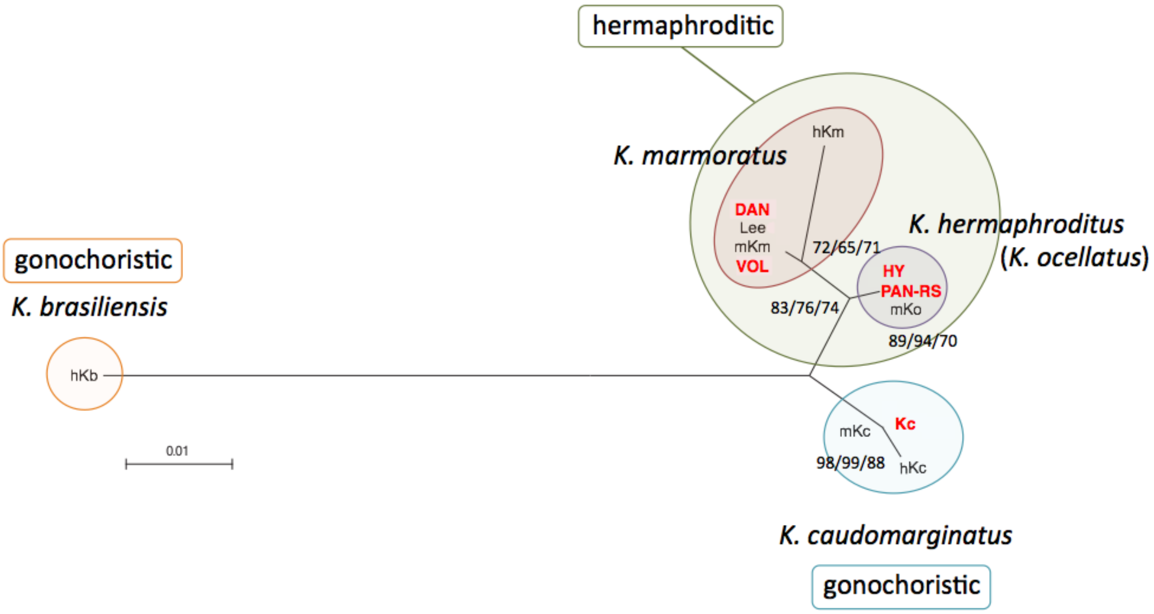

Figure S1. Phylogenetic relationship of hermaphroditic *Kryptolebias marmoratus* and *K. hermaphroditus* strains and a sister gonochoristic species, *K. caudomargimatus* with an outgroup, *K. brasiliensis*. The tree was constructed by neighbor-joining (NJ) method based on mitochondrial DNA sequences (a part of 12S rRNA, corresponding to nucleotide 17 to 332 in KP998194). Tree topology did not change when analyzed with maximum likelihood (ML) or maximum parsimony (MP) methods (data not shown). Numbers at the nodes (NJ/ML/MP) represent percentage recovery of those nodes per 10,000 bootstrap replicates. Only values above 60% are shown. The data from the present study are indicated in bold red. The hermaphroditic mangrove killifish is clearly separated into two groups, *K. marmoratus* and *K. hermaphroditus* (formerly *K. ocellatus*). The accession numbers are: hKb (AY946281) and hKm (AY946280) (Vermeulen and Hrbek, 2005); hKc (AF092294, Hrbek and Larson, 1999); mKc (AF002428), mKm (AF002429), and mKo (AF002430) (Murphy *et al*. 1999); Lee, Km (AF283503, Lee *et al*. 2001). Scale bars indicate genetic distance.
