# Supplementary material for: A Genetic Map for the Only Self-Fertilizing Vertebrate
Source: G3 (Bethesda). 2016 Feb 9;6(4):1095–106. doi: 10.1534/g3.115.022699 (PMC4825644; doi:10.1534/g3.115.022699)
Supplement: Supplemental Material [file supp_g3.115.022699_FigureS2.pdf]

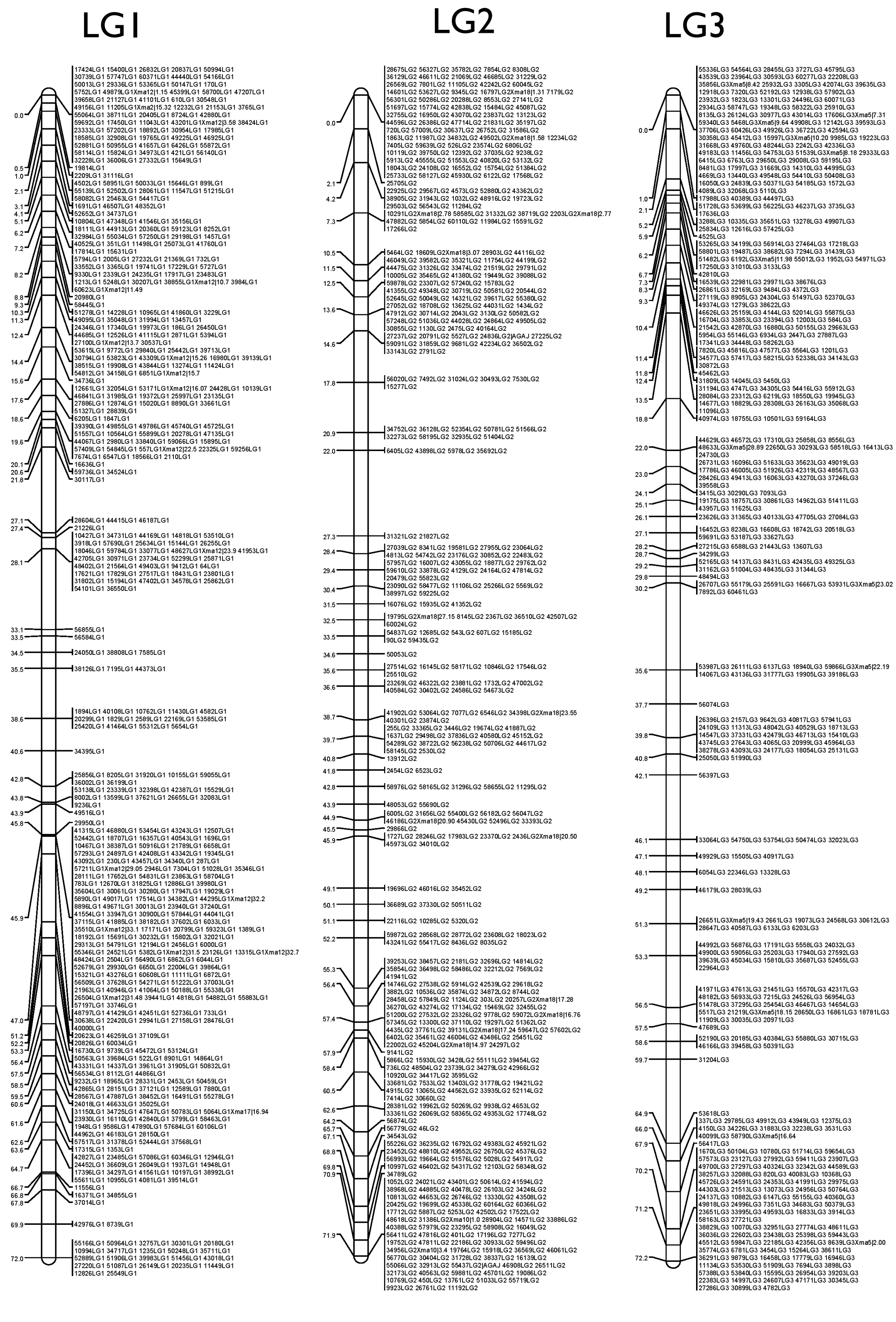
LG1
LG2
LG3

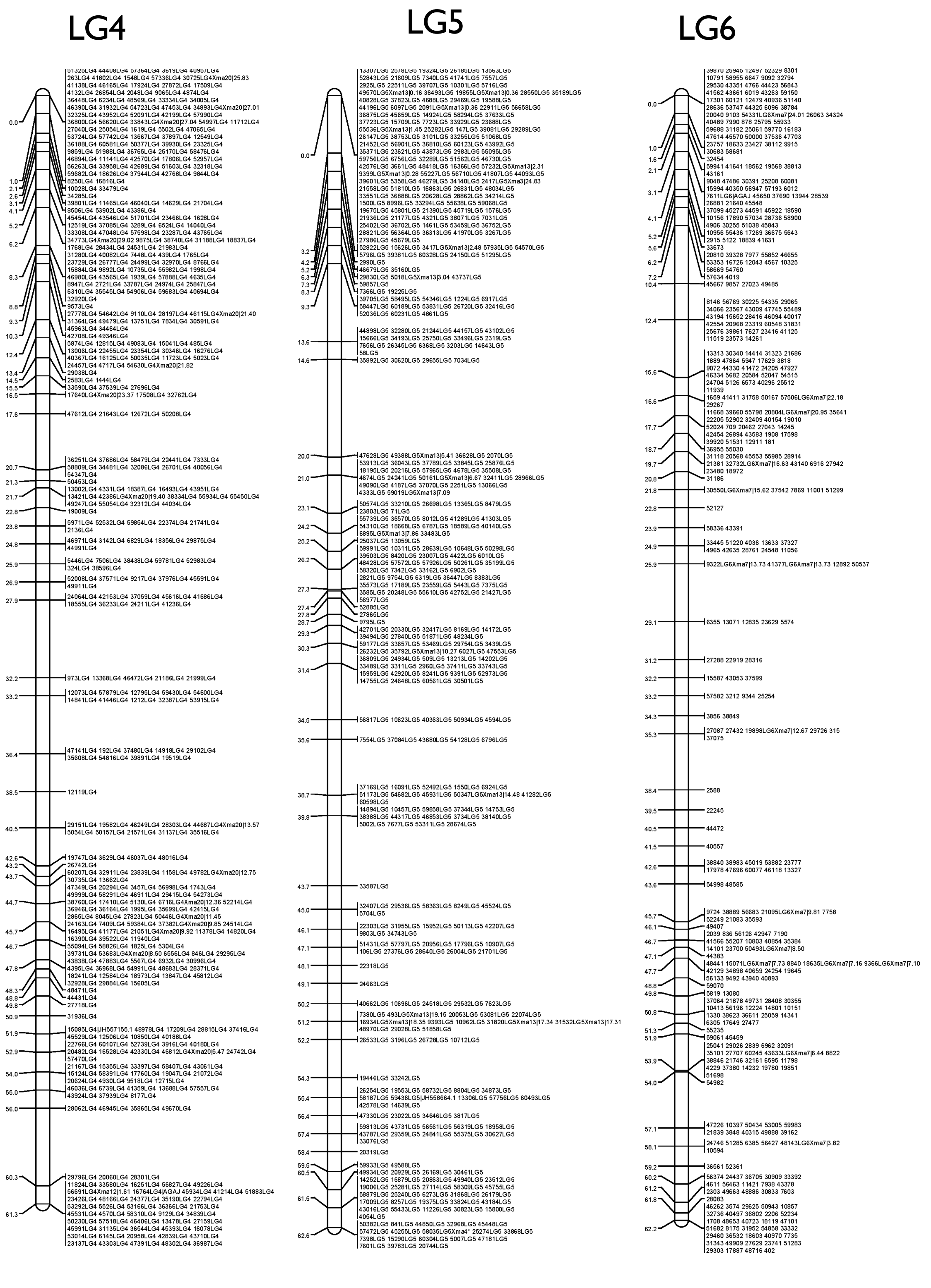
LG4
LG5
LG6

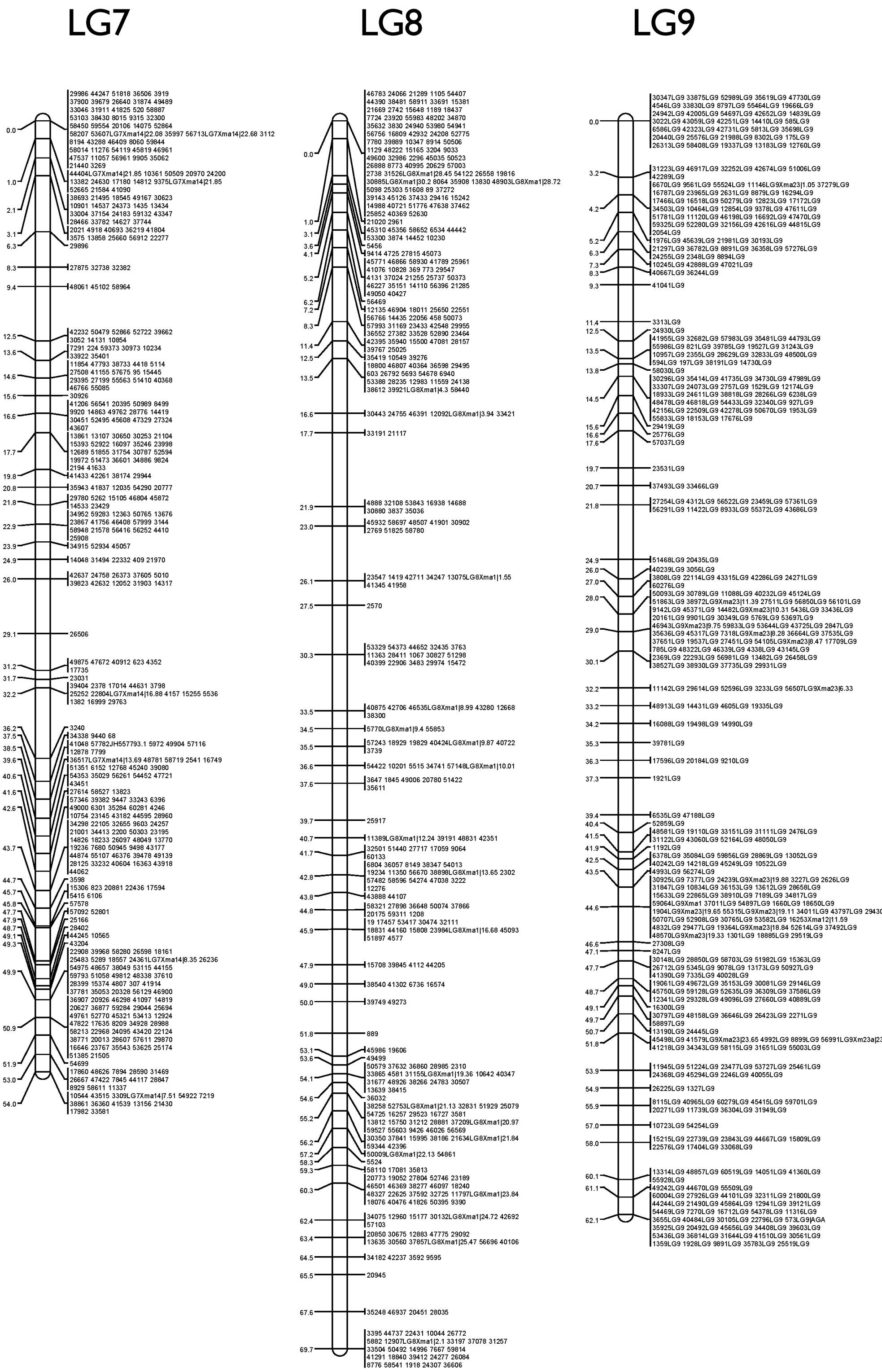

LG7
LG8
LG9

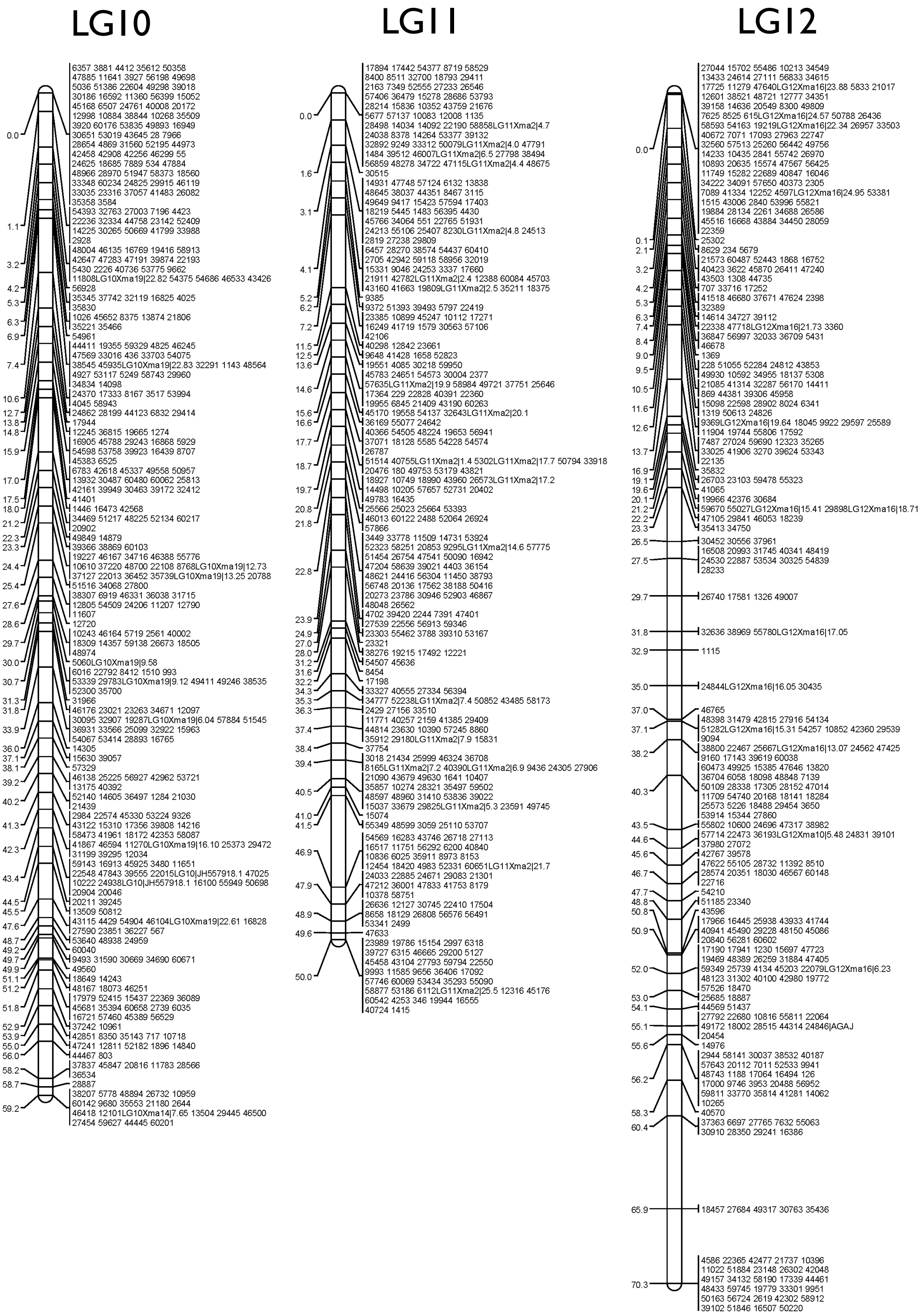
LG10
LG11
LG12

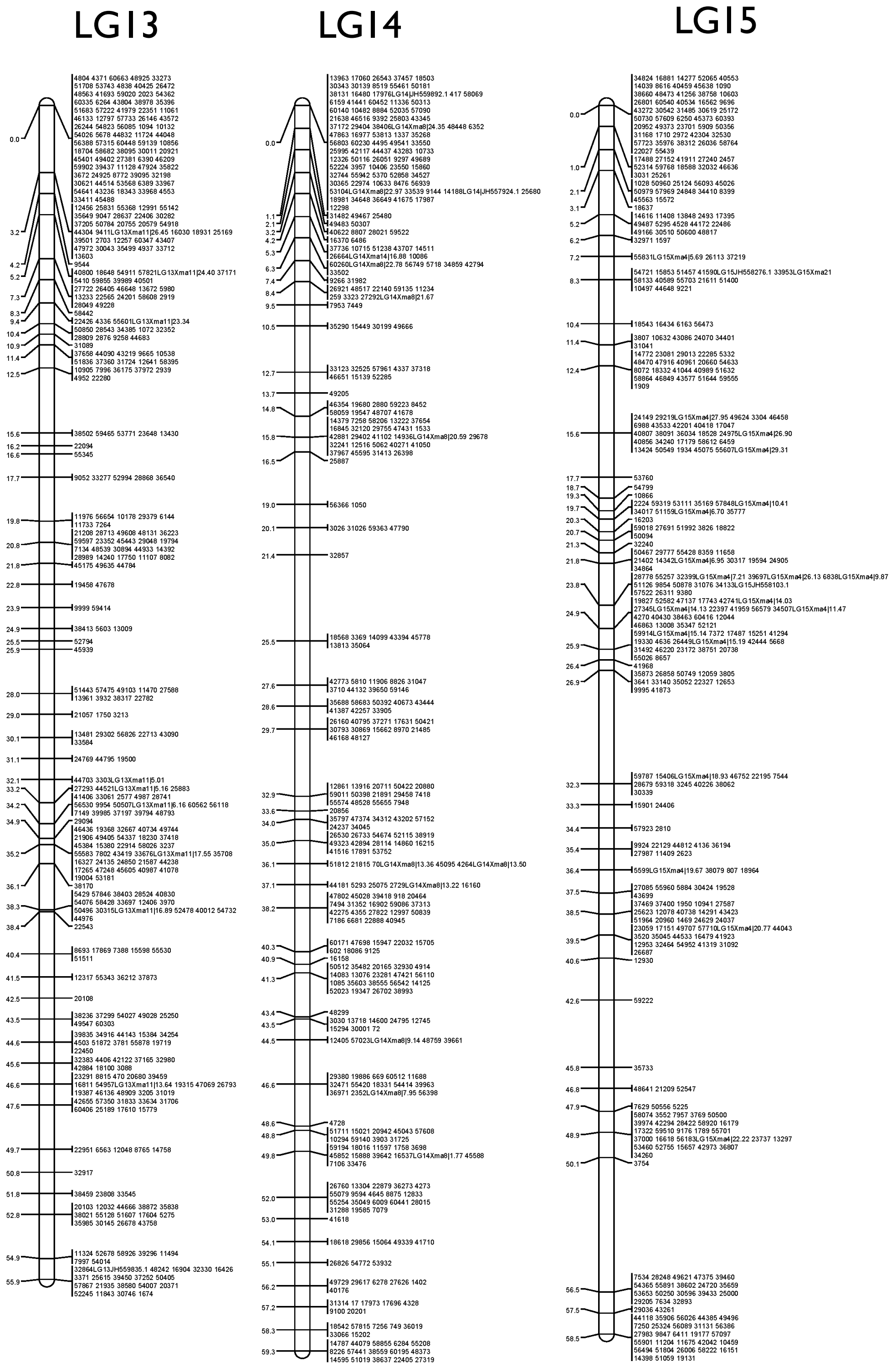
LG13
LG14
LG15

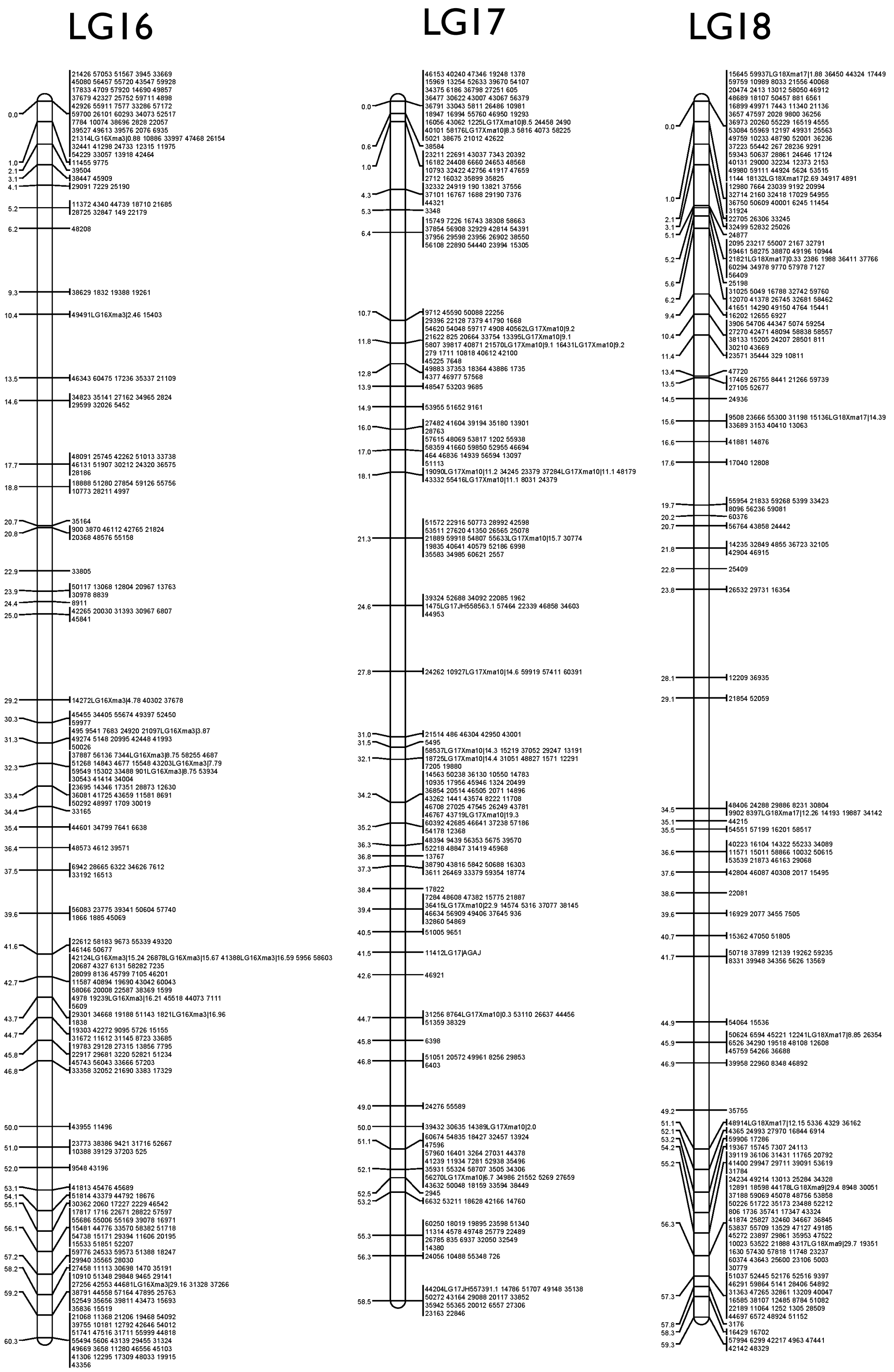
LG16
LG17
LG18

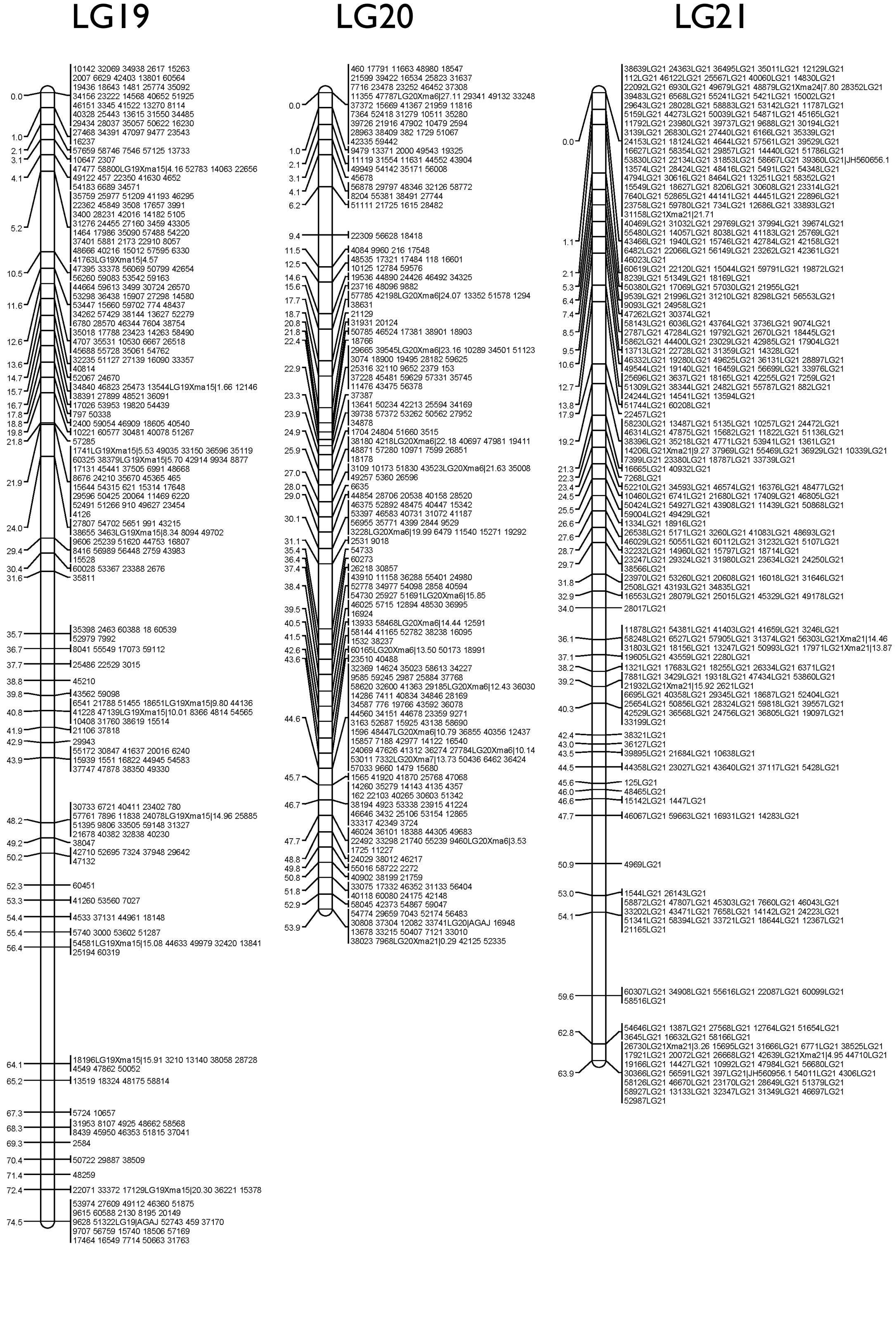
LG19
LG20
LG21

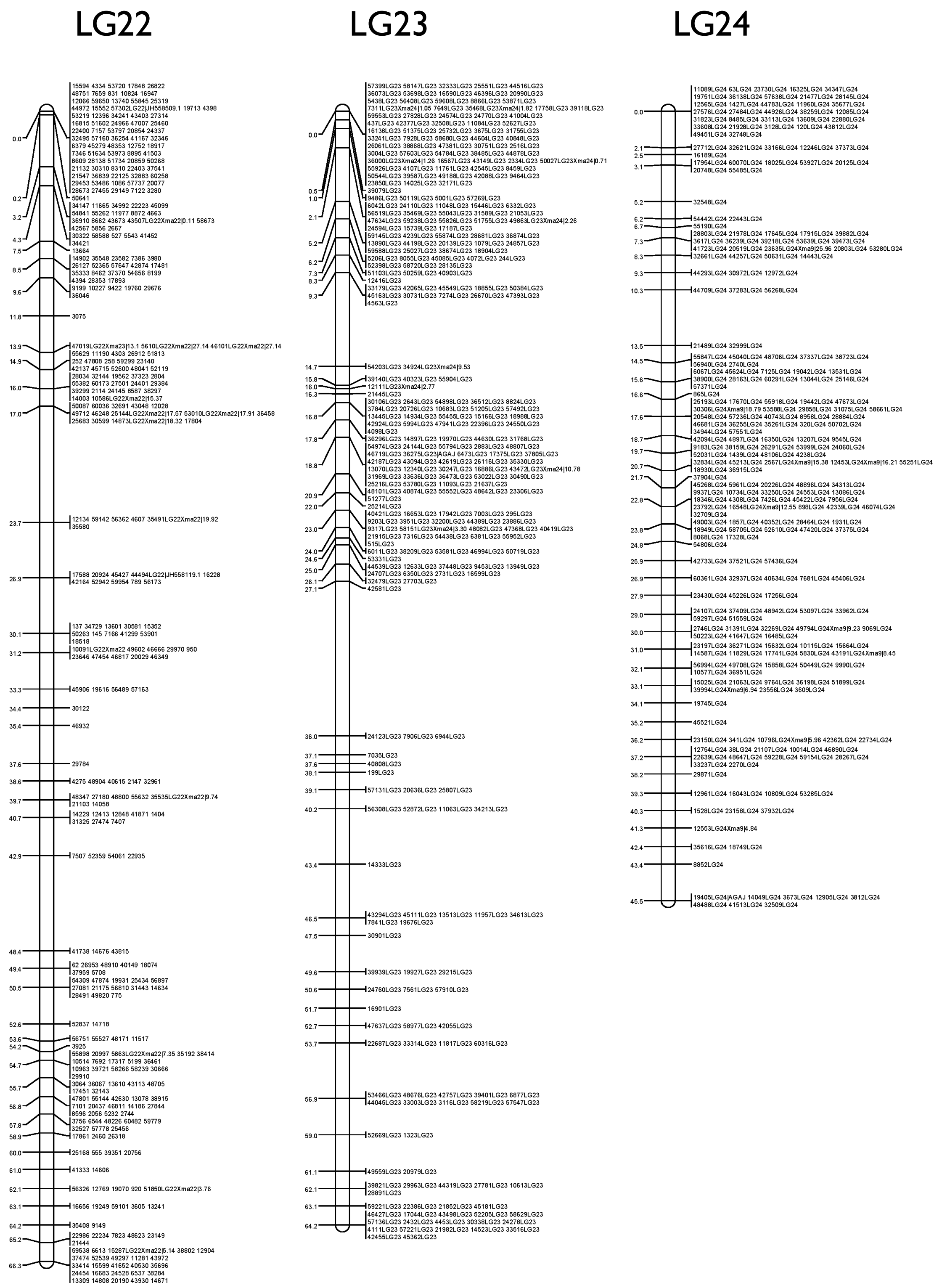
LG22
LG23
LG24

Figure S2. A Genetic linkage map of *Kryptplebias marmoratus*/*K. hermaphroditus* constructed with JoinMap 4.1. We obtained identical marker grouping into LGs as the PHP program and marker ordering consistent with the PHP program as shown in Figure 2.
