# Supplementary material for: A Genetic Map for the Only Self-Fertilizing Vertebrate
Source: G3 (Bethesda). 2016 Feb 9;6(4):1095–106. doi: 10.1534/g3.115.022699 (PMC4825644; doi:10.1534/g3.115.022699)
Supplement: Supplemental Material [file supp_g3.115.022699_FigureS3.pdf]

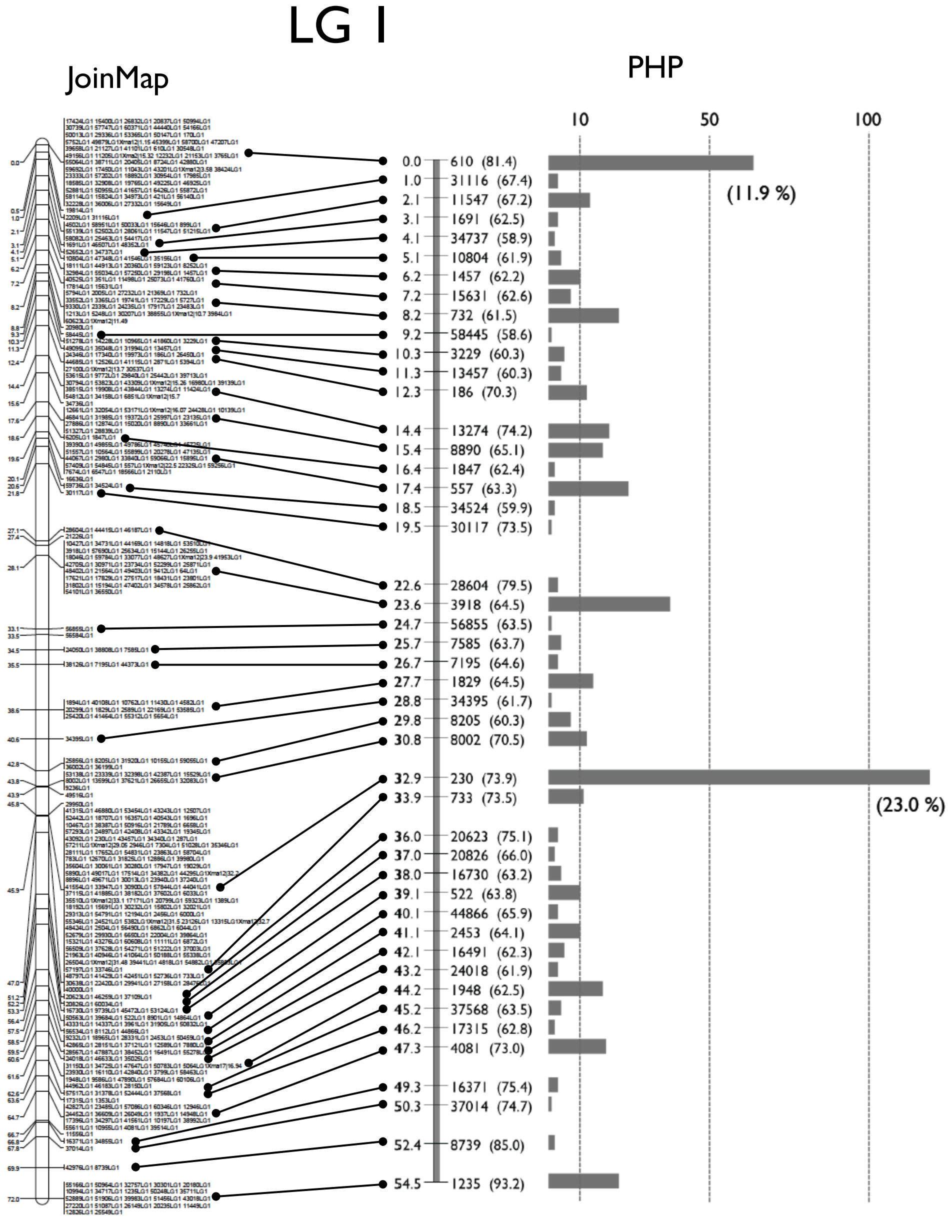

Figure S3. A comparison of LG1 linkage maps of *Kryptplebias marmoratus*/*K. hermaphroditus* constructed with either JoinMap 4.1 or the PHP programs. The marker grouping and locus ordering are consistent in both programs. However, the distances between markers were calculated differently in some cases because the algorithms used in two programs were distinct.
