# Supplementary material for: A Genetic Map for the Only Self-Fertilizing Vertebrate
Source: G3 (Bethesda). 2016 Feb 9;6(4):1095–106. doi: 10.1534/g3.115.022699 (PMC4825644; doi:10.1534/g3.115.022699)
Supplement: Supplemental Material [file supp_g3.115.022699_FigureS4.pdf]

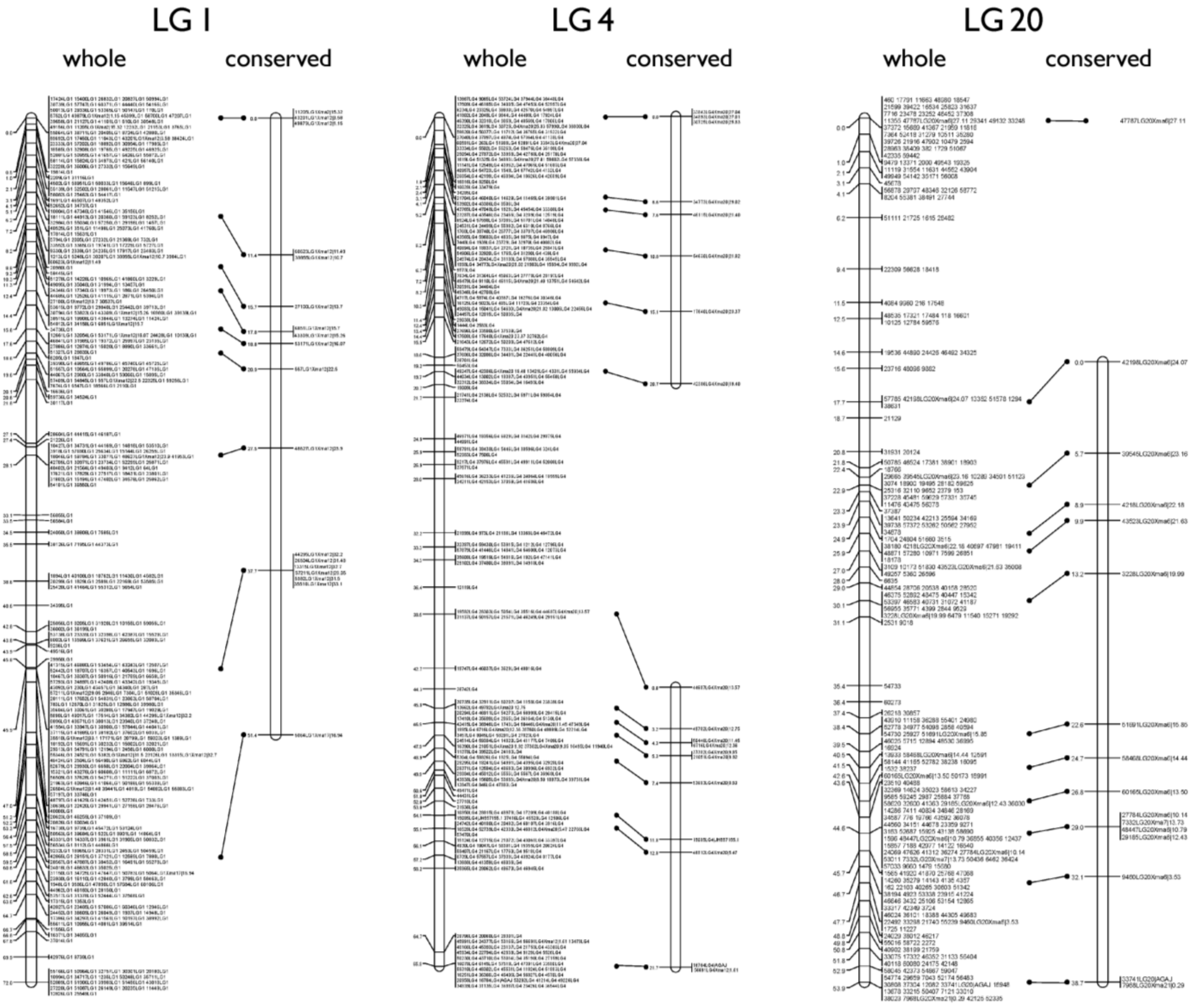

Figure S4. A comparison of genetic maps of *Kryptplebias marmoratus*/*K. hermaphroditus* constructed with a whole set of markers (whole) and with a subset of markers whose sequences showed conservations with platyfish (conserved). Marker grouping and marker ordering are consistent in both maps. For 18 LGs, the markers were grouped together and were ordered identically (e.g. LG1). The results corroborate the ordering obtained in the whole map. The exceptions were LG4 (together with LGs 7 and 13) where the markers were in two groups or LG20 (together with LGs 9 and 21) where most markers were in one group with a single marker not linked to those.
