# Supplementary material for: A Genetic Map for the Only Self-Fertilizing Vertebrate
Source: G3 (Bethesda). 2016 Feb 9;6(4):1095–106. doi: 10.1534/g3.115.022699 (PMC4825644; doi:10.1534/g3.115.022699)
Supplement: Supplemental Material [file supp_g3.115.022699_FigureS5.pdf]

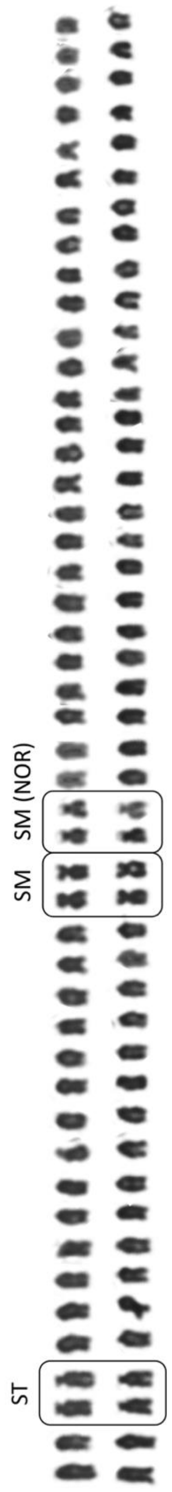

Figure S5. Metaphase chromosomes of *Kryptolebias hermaphroditus* obtained from primary cultured fibroblasts of a HY strain adult. Two sets of representative Giemsa stained samples shown were composed of 48 chromosomes. Total of 23 spreads were counted and 48 was a mode (data not shown). Except for one pair of subtelocentric (ST) and two pairs of submetacentric (SM) chromosomes, all were acrocentric chromosomes gradually changing in size. One pair of SM chromosomes had less stained areas in their short arms, probably representing a nucleolus organizing region (NOR).
