# Supplementary material for: A Genetic Map for the Only Self-Fertilizing Vertebrate
Source: G3 (Bethesda). 2016 Feb 9;6(4):1095–106. doi: 10.1534/g3.115.022699 (PMC4825644; doi:10.1534/g3.115.022699)
Supplement: Supplemental Material [file supp_g3.115.022699_FigureS6.pdf]

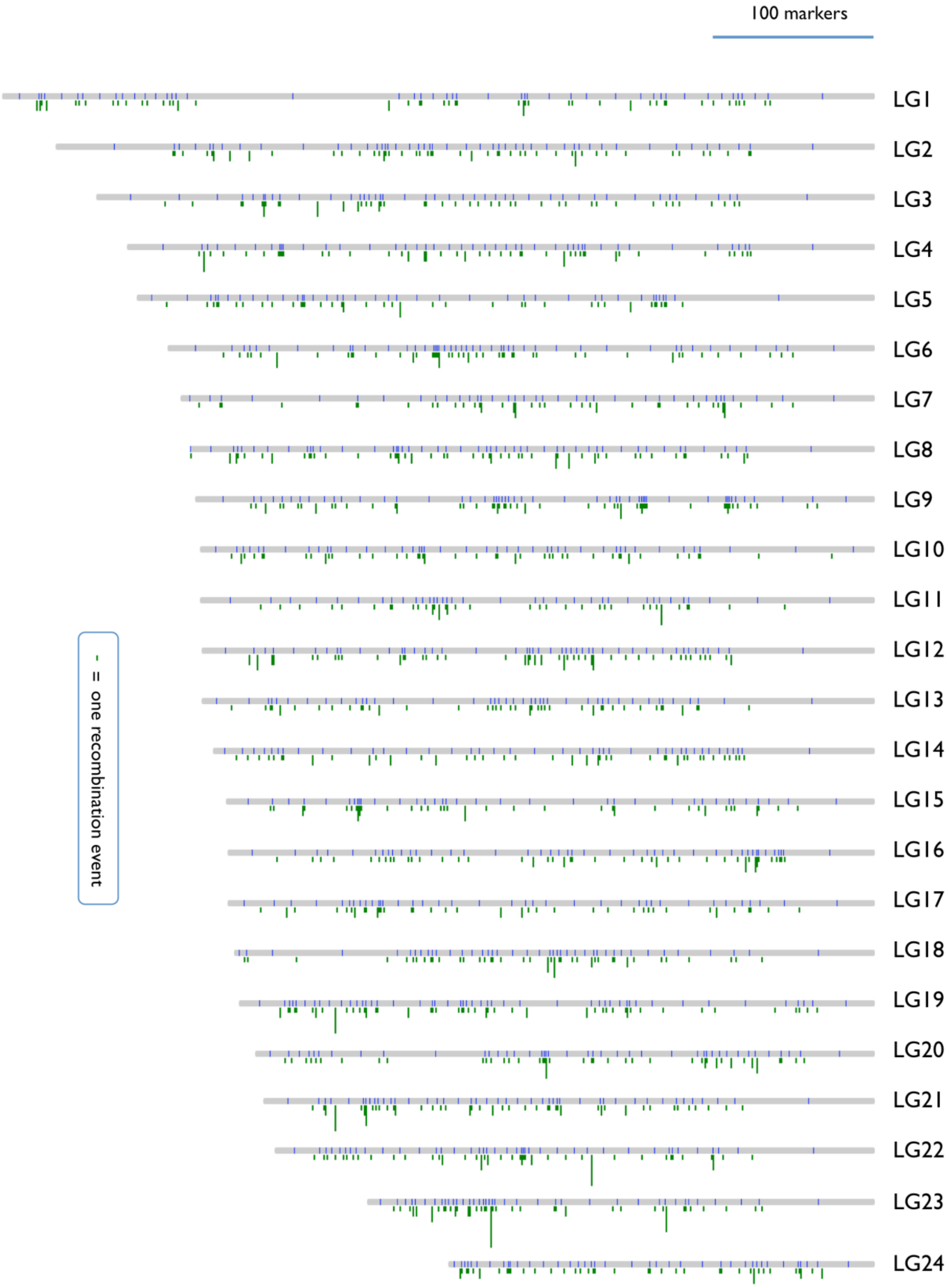
100 markers
LG1
LG2
LG3
LG4
LG5
LG6
LG7
LG8
LG9
LG10
LG11
LG12
LG13
LG14
LG15
LG16
LG17
LG18
LG19
LG20
LG21
LG22
LG23
LG24
| = one recombination event

Figure S6. A Genetic linkage map of *Kryptplebias marmoratus*/*K. hermaphroditus* based on RAD sequencing. The map was coalesced into 24 linkage groups (LGs). LGs were named in decreasing order of total number of markers, relative to which each vertical bar (grey) was drawn. Each bin (location of co-segregating markers) occupies length relative to total number of co-segregating markers, middle of which blue horizontal lines were plotted. Green horizontal bars, drawn on the right of the grey bars, represent numbers of recombination events and were plotted between adjacent bins. Assuming relatively even distribution of markers, these grey bars represent physical maps with density of green bars as relative frequency of recombination events along the chromosomes.
