# Supplementary material for: A Genetic Map for the Only Self-Fertilizing Vertebrate
Source: G3 (Bethesda). 2016 Feb 9;6(4):1095–106. doi: 10.1534/g3.115.022699 (PMC4825644; doi:10.1534/g3.115.022699)
Supplement: Supplemental Material [file supp_g3.115.022699_FigureS7.pdf]

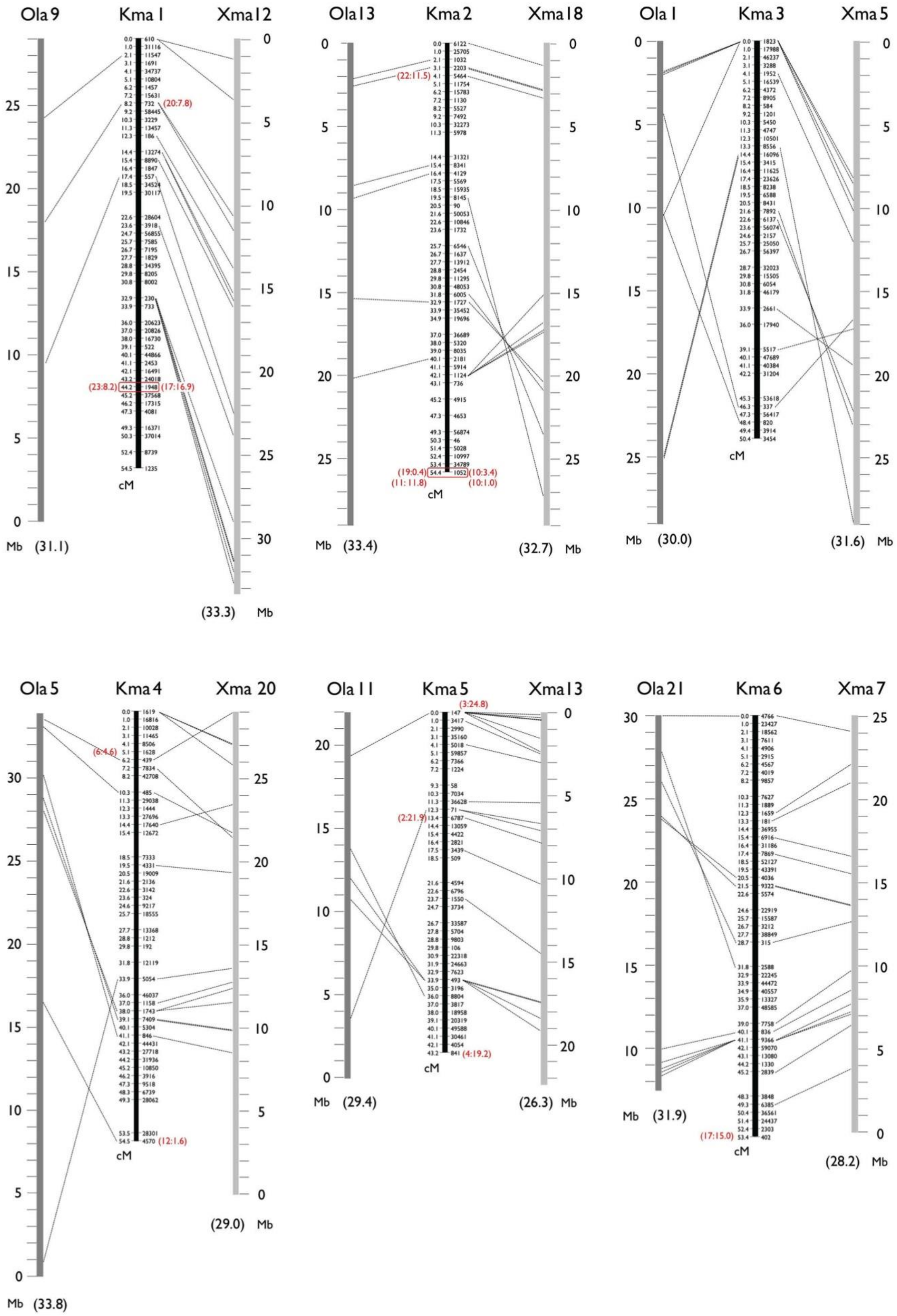

Ola 9
Kma 1
Xma 12
Mb (31.1)
cM
(33.3) Mb
(20:7.8)
(23:8.2)
(17:16.9)
Ola 13
Kma 2
Xma 18
Mb (33.4)
(32.7) Mb
cM
(22:11.5)
(19:0.4)
(11:11.8)
(10:3.4)
(10:1.0)
Ola 1
Kma 3
Xma 5
Mb (30.0)
(31.6) Mb
cM
Ola 5
Kma 4
Xma 20
Mb (33.8)
(29.0) Mb
cM
(6:4.6)
(12:1.6)
Ola 11
Kma 5
Xma 13
Mb (29.4)
(26.3) Mb
cM
(3:24.8)
(2:21.9)
(4:19.2)
Ola 21
Kma 6
Xma 7
Mb (31.9)
(28.2) Mb
cM
(17:15.0)

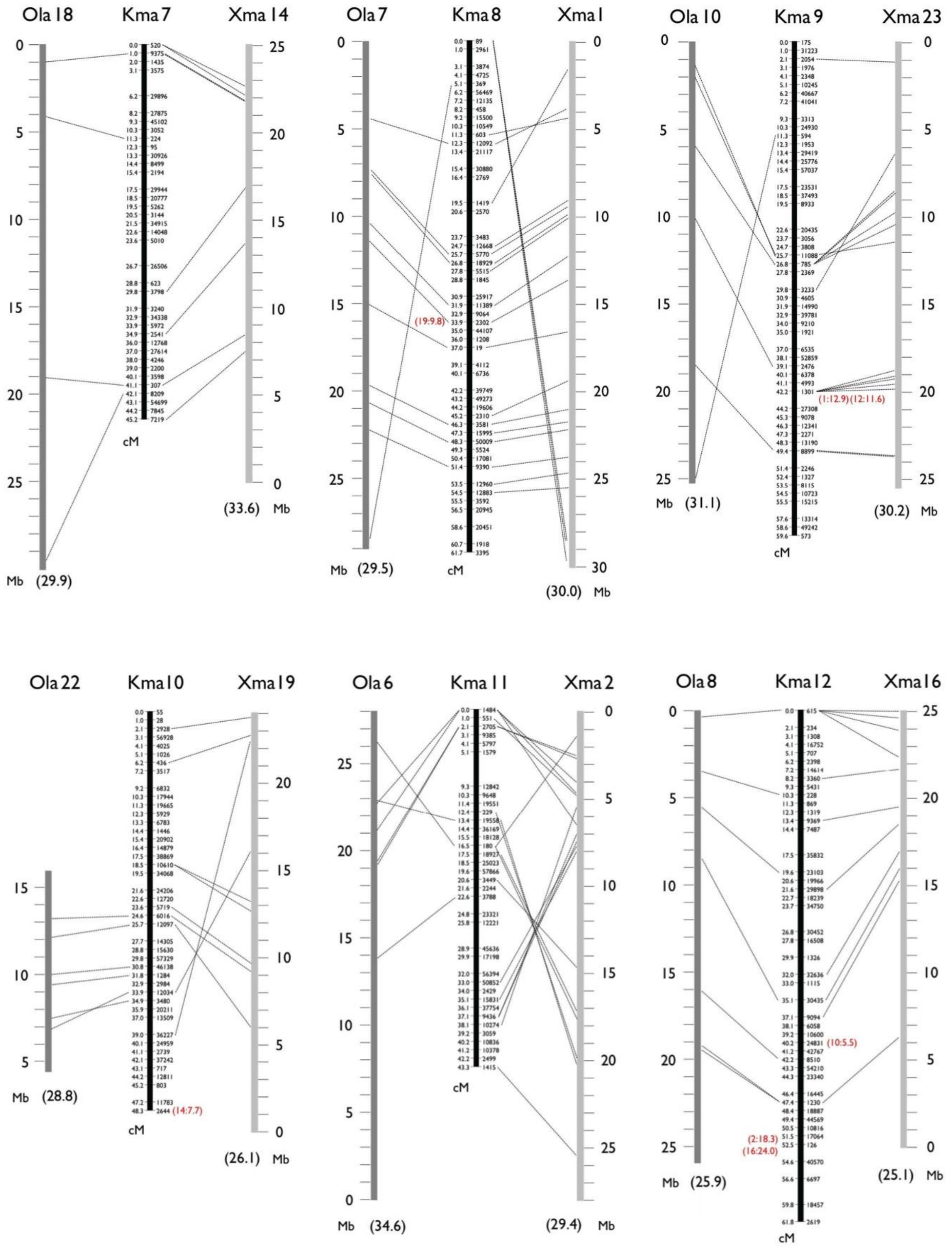

Ola 18
Kma 7
Xma 14
Mb (29.9)
cM
(33.6) Mb
Ola 7
Kma 8
Xma 1
(19:9.8)
Mb (29.5)
cM
(30.0) Mb
Ola 10
Kma 9
Xma 23
(1:12.9) (12:11.6)
Mb (31.1)
cM
(30.2) Mb
Ola 22
Kma 10
Xma 19
(14:7.7)
Mb (28.8)
cM
(26.1) Mb
Ola 6
Kma 11
Xma 2
Mb (34.6)
cM
(29.4) Mb
Ola 8
Kma 12
Xma 16
(10:5.5)
(2:18.3)
(16:24.0)
Mb (25.9)
cM
(25.1) Mb

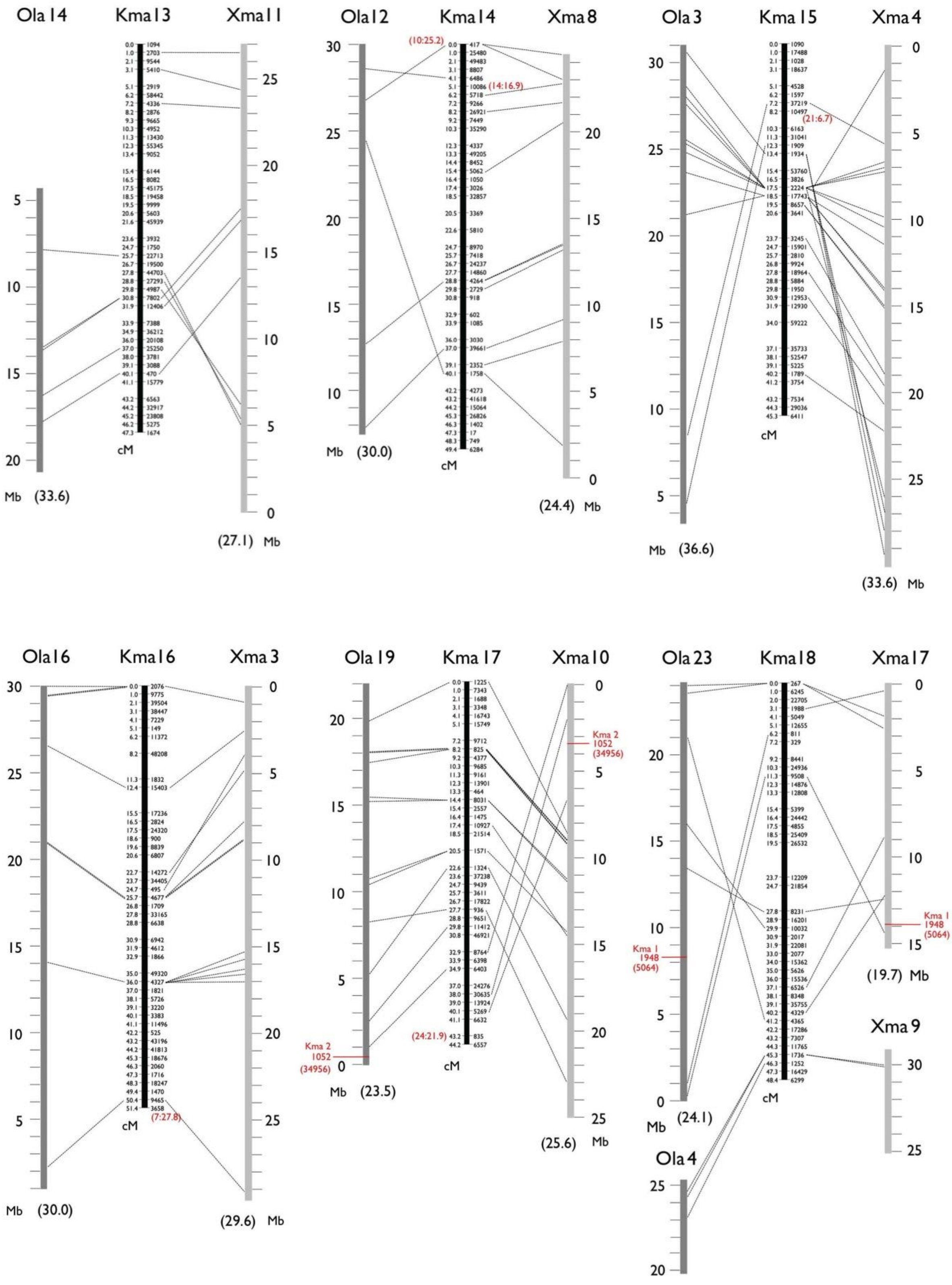

Ola 14
Kma13
Xma11
Mb (33.6)
cM
(27.1) Mb
Ola12
Kma14
Xma 8
(10:25.2)
(14:16.9)
Mb (30.0)
cM
(24.4) Mb
Ola 3
Kma 15
Xma 4
(21:6.7)
Mb (36.6)
cM
(33.6) Mb
Ola16
Kma16
Xma 3
(7:27.8)
Mb (30.0)
cM
(29.6) Mb
Ola 19
Kma 17
Xma10
Kma 2
1052
(34956)
(24:21.9)
Mb (23.5)
cM
(25.6) Mb
Ola 23
Kma18
Xma17
Kma 1
1948
(5064)
Mb (24.1)
cM
(19.7) Mb
Xma 9
Ola 4

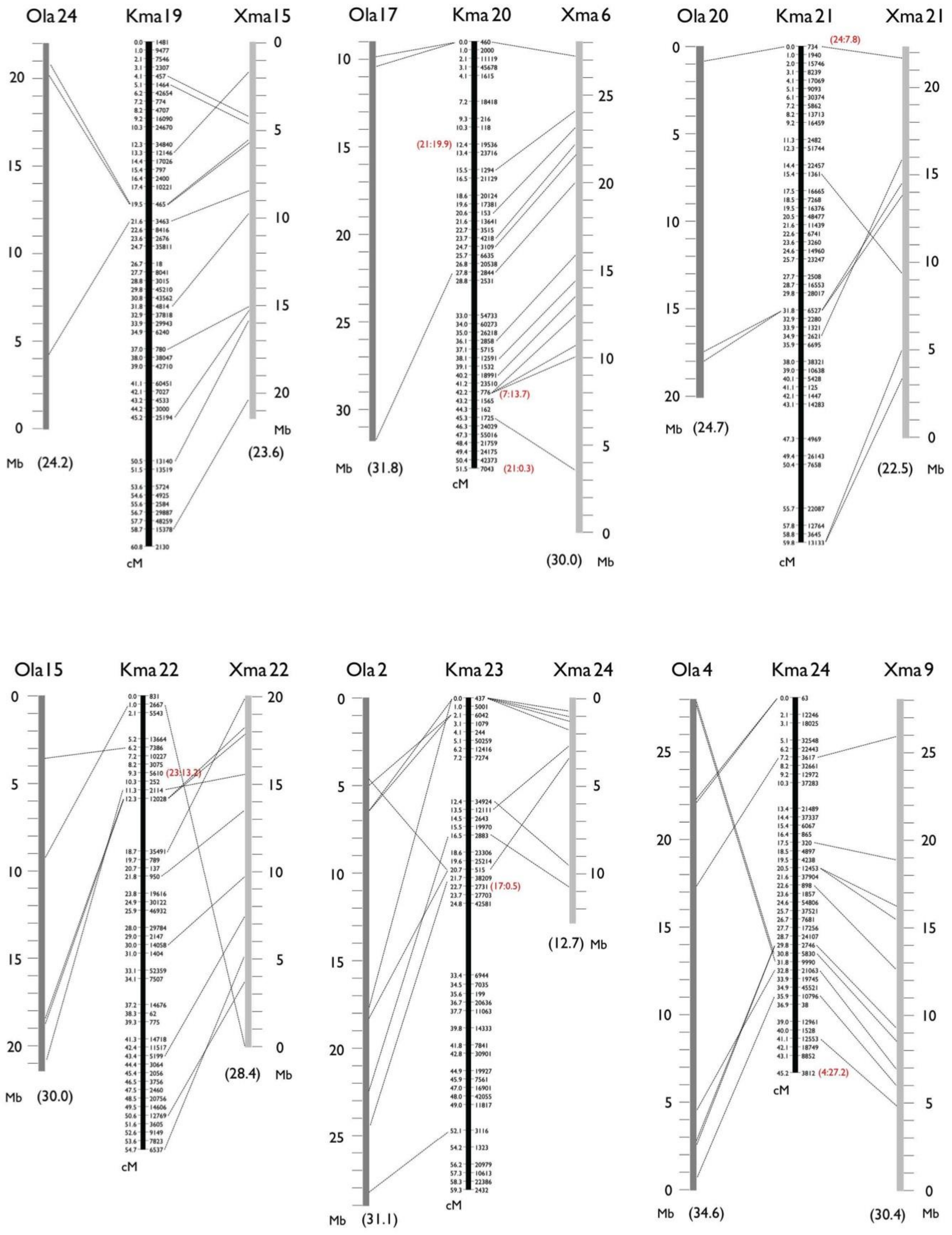

Ola 24
Kma 19
Xma 15
Mb
(24.2)
(23.6)
cM
Ola 17
Kma 20
Xma 6
(21:19.9)
(7:13.7)
(21:0.3)
Mb
(31.8)
(30.0)
Ola 20
Kma 21
Xma 21
(24:7.8)
Mb
(24.7)
(22.5)
Ola 15
Kma 22
Xma 22
(23:13.2)
(30.0)
(28.4)
Ola 2
Kma 23
Xma 24
(17:0.5)
(12.7)
(31.1)
Ola 4
Kma 24
Xma 9
(4:27.2)
(34.6)
(30.4)

Figure S7. Conserved synteny of *Kryptolebias marmoratus*/*K. hermaphroditus* linkage groups (Kma LGs) to either platyfish (*Xiphophorus maculatus*, Xma) or medaka (*Oryzias latipes*, Ola) chromosomes, suggesting basically one to one relationship (24 orthologous chromosome pairs). Dotted lines indicate homology of mapped Kma markers to either Xma or Ola mapped genome sequences identified by the blastn program with default parameters. For platyfish, blast hits with a cut-off e-value of 1.0E-9 are shown. Many Kma LGs show intrachromosomal rearrangements probably due to recurred inversions after divergence from common ancestors between either Xma or Ola. Some Kma markers show homology to sequences on non-orthologous chromosomes (indicated by red letters in parenthesis). For example, a marker at 8.2 cM on Kma LG1 (Kma1) has a homology to a sequence on Xma chromosome 20 at 7.8 Mb. Two such markers, 5064 (represented by 1948 on Kma LG1) and 34956 (represented by 1052 on Kma LG2), had homologous sequences on another orthologous chromosomes (Ola23-Kma18-Xma17 and Ola19-Kma17-Xma10, respectively), indicating translocations happened after the *K. marmoratus*/*K. hermaphroditus* lineage diverged from platyfish. Similarly, markers 18609 (represented by 5464 on Kma LG2) and 38898 (represented by 2302 on Kma LG8), had homologous sequences on orthologous platy chromosomes (Xma18 and Xma1, respectively) but on non-orthologous medaka chromosomes (Ola22 and Ola19, respectively), indicating translocations happened after the medaka lineage diverged from cyprinodontiformes.
