# Supplementary material for: A Genetic Map for the Only Self-Fertilizing Vertebrate
Source: G3 (Bethesda). 2016 Feb 9;6(4):1095–106. doi: 10.1534/g3.115.022699 (PMC4825644; doi:10.1534/g3.115.022699)
Supplement: Supplemental Material [file supp_g3.115.022699_TableS1.pdf]

Table S1. Number of tags classified by presence (1) or absence (0) in each sample.

	K. marmoratus		*K.hermaphroditus*			
sample	DAN	VOL	PAN-RS	HY	Kc	number of tags
	1	1	1	1	1	35415
	0	1	1	1	1	98
	1	0	1	1	1	116
	1	1	0	1	1	170
	1	1	1	0	1	212
	1	1	1	1	0	16426
	0	0	1	1	1	672
	0	1	0	1	1	8
	0	1	1	0	1	5
	0	1	1	1	0	132
	1	0	0	1	1	4
	1	0	1	0	1	7
	1	0	1	1	0	162
	1	1	0	0	1	489
	1	1	0	1	0	235
	1	1	1	0	0	279
	0	0	0	1	1	72
	0	0	1	0	1	49
	0	0	1	1	0	1752
	0	1	0	0	1	38
	0	1	0	1	0	33
	0	1	1	0	0	22
	1	0	0	0	1	22
	1	0	0	1	0	29
	1	0	1	0	0	45
	1	1	0	0	0	1975
	1	0	0	0	0	347
	0	1	0	0	0	391
	0	0	1	0	0	646
	0	0	0	1	0	717
	0	0	0	0	1	18679
total	57214	57217	57489	57361	56859	79247

(35415 tags:) identical 9240
polymorphic 26175
(total SNPs 53544)
