# Supplementary material for: A Genetic Map for the Only Self-Fertilizing Vertebrate
Source: G3 (Bethesda). 2016 Feb 9;6(4):1095–106. doi: 10.1534/g3.115.022699 (PMC4825644; doi:10.1534/g3.115.022699)

Outline of the mapping program (linkage.class.php)

Step1. Sort unique markers (binning)

Marker	Types (genotype patterns for 49 F_2 individuals)	Number of missing genotypes (0–7)
63	h h b h h b a h a b … a a a b b h a a	0
120	h h b h h b a h a b … a a a b b h a a	0
3128	h h b h h b a h a b … a a - b - h a a	2
21477	h h b h h b a h a b … - - a - b h a a	3

For example, these four markers above were binned together as "b63" (b + representative marker 63). Missing genotypes were removed and remaining genotype patterns were used for sorting. If a marker could belong to two bins (116 markers) or three bins (3 markers), it was arbitrarily assigned to one of the bins. Genotype symbols represent following: a, DAN homozygous; b, PAN-RS homozygous; h, heterozygous; -, missing genotype.

Step2. Make distance matrix

		Bin				
		b803	b2644	b11783	b12811	b36534
Bin	b717	2/96	6/98	5/98	1/98	4/98
	b803		4/96	3/96	1/96	2/96
	b2644			1/98	5/98	2/98
	b11783				4/98	1/98
	b12811					3/98

The distances among all bins were calculated as follows. Changes between homozygous (a or b) to heterozygous (h) were scored as one recombination event (1) and changes between homozygous (a to b) as two (2). The distances were calculated as sum of these recombination scores / 2X number of F_2 without missing genotypes (number of total meioses; 98 if all 49 F2 had homozygous or heterozygous genotypes for both markers in comparison and 96 if one of the marker, such as b803 above, had one missing genotype). An initial distance matrix was constructed for all bins (1,157 in the present study).

Step3. Join neighboring markers into linkage groups

3-1. Pick one of the nearest pair (having smallest distance in the matrix) from the distance matrix (*e.g.* b2644 and b11783 shown above).
3-2. Make linkage group "LG2644" (LG + representative bin) and remove b11783 from the initial matrix.
3-3. The smaller distance of the two is chosen as a new distance for "LG2644". LGs always retain information on which bin is the nearer to each remaining bin (in this case, b11783 is nearer to all remaining bins).

	b2644	b11783	LG2644
b717	6/98	5/98	5/98
b803	4/96	3/96	3/96
b12811	5/98	4/98	4/98
b36534	2/98	1/98	1/98

The matrix is now changed as follows.

		Bin/LG			
		b803	LG2644	b12811	b36534
	b717	2/96	5/98	1/98	4/98
Bin/LG	b803		3/96	1/96	2/96
	LG2644			4/98	1/98
	b12811				3/98

3-4. Back to 3-1 and repeat linkage grouping until remaining distances in the matrix reach maximum recombination rate (a parameter set in linkage.class.php script; 0.15 was chosen in the present map). For this example matrix, b36534 is next joined to LG2644, which now contains locus ordering and distance information as below.

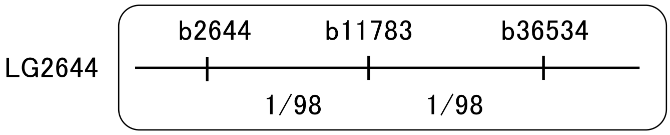

In this way, linkage grouping and bin ordering proceed simultaneously.
Distances are calculated again as follows.

	b2644	b36534	LG2644
b717	6/98	4/98	4/98
b803	4/96	2/96	2/96
b12811	5/98	3/98	3/98

New matrix again.

		Bin/LG		
		b803	LG2644	b12811
	b717	2/96	4/98	1/98
Bin/LG	b803		2/96	1/96
	LG2644			3/98

Next, b717 and b12811 are joined as LG717.　New distances and matrix again.

	b717	b12811	LG717
b803	2/96	1/96	1/96
LG2644	4/98	3/98	3/98

		Bin/LG	
		b803	LG2644
Bin/LG	LG717	1/96	3/98
	b803		2/96

Join LG717 and b803.　A new distance is calculated.

	b717	b803	LG717
LG2644	4/98	2/98	2/98

Now LG 717 contains following grouping and bin ordering.

LG717

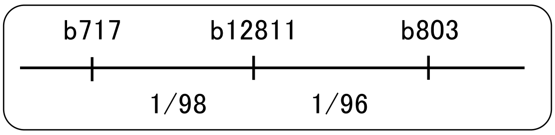

The distance between LG717 and LG2644 is 2/98, which is a distance between b803 and b36534.　Therefore the initial bins are all joined as a final LG shown below.

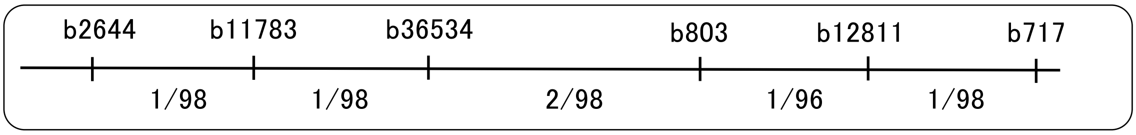

Supplement: Supplemental Material [file supp_g3.115.022699_FileS3.zip › MappingOutline.pdf]
